# Differential and Conditional Activation of PKC-Isoforms Dictates Cardiac Adaptation during Physiological to Pathological Hypertrophy

**DOI:** 10.1371/journal.pone.0104711

**Published:** 2014-08-12

**Authors:** Shaon Naskar, Kaberi Datta, Arkadeep Mitra, Kanchan Pathak, Ritwik Datta, Trisha Bansal, Sagartirtha Sarkar

**Affiliations:** Genetics and Molecular Cardiology Laboratory, Department of Zoology, University of Calcutta, Kolkata, West Bengal, India; Texas A & M, Division of Cardiology, United States of America

## Abstract

A cardiac hypertrophy is defined as an increase in heart mass which may either be beneficial (physiological hypertrophy) or detrimental (pathological hypertrophy). This study was undertaken to establish the role of different protein kinase-C (PKC) isoforms in the regulation of cardiac adaptation during two types of cardiac hypertrophy. Phosphorylation of specific PKC-isoforms and expression of their downstream proteins were studied during physiological and pathological hypertrophy in 24 week male Balb/c mice (*Mus musculus*) models, by reverse transcriptase-PCR, western blot analysis and M-mode echocardiography for cardiac function analysis. PKC-δ was significantly induced during pathological hypertrophy while PKC-α was exclusively activated during physiological hypertrophy in our study. PKC-δ activation during pathological hypertrophy resulted in cardiomyocyte apoptosis leading to compromised cardiac function and on the other hand, activation of PKC-α during physiological hypertrophy promoted cardiomyocyte growth but down regulated cellular apoptotic load resulting in improved cardiac function. Reversal in PKC-isoform with induced activation of PKC-δ and simultaneous inhibition of phospho-PKC-α resulted in an efficient myocardium to deteriorate considerably resulting in compromised cardiac function during physiological hypertrophy via augmentation of apoptotic and fibrotic load. This is the first report where PKC-α and -δ have been shown to play crucial role in cardiac adaptation during physiological and pathological hypertrophy respectively thereby rendering compromised cardiac function to an otherwise efficient heart by conditional reversal of their activation.

## Introduction

Cardiac hypertrophy is defined as an increase in cardiac mass and two forms of cardiac hypertrophy viz. physiological and pathological are currently recognized. Physiological increase in cardiac mass is considered to be an adaptive beneficial response which occurs normally during development, pregnancy and in response to sustained exercise [1] that enhances normal cardiac structure and normal or improved cardiac function with no evidence of myocyte apoptosis [2], [3]. In contrast, pathological cardiac hypertrophy occurring in response to diverse stimuli including hypertension, valve disease, genetic mutations etc. [4] is detrimental resulting in loss of cardiac function, accumulation of collagen, loss of cardiomyocytes and ultimately heart failure [5].

Among various signaling pathways involved in promoting cardiac hypertrophy protein kinase-C (PKC) has been identified as an important component used by myocytes in response to a variety of extracellular stimuli [6]. PKC, a class of phospholipid dependent serine/threonine kinase, is activated by second messenger molecules formed via receptor-dependent activation of phospholipase-C. Cardiomyocytes express multiple PKC-isoforms with distinct biological functions in different cardiac diseases [7], [8]. PKC-α is involved in the development of cardiac hypertrophy though extracellular-signal-regulated kinase-1/2 (ERK-1/2) [9] whereas, activation of PKC-ε has positive effect on cell growth during ventricular hypertrophy [10]. PKC-ε has also been shown to protect post-conditioned cardiomyocytes from programmed cell death [11]. Several reports have shown involvement of PKC-α, -β, and -δ in development of cardiac hypertrophy [7, 8 &12]. Increased PKC-δ expression and decreased expression of PKC-α, -β, -ε, and -ζ was observed in aortic banding model of cardiac hypertrophy [13]. PKC-isoforms have been shown to have either pro (PKC-δ) or anti (PKC-λ, -ζ and -α) apoptotic function in different cell types [14].

Several researchers have shown cardio-protective role of regular exercise in individuals suffering from cardiovascular diseases [15] and endurance training could convert pathological cardiac hypertrophy into physiological form with improved cardiac performances [16]. Although a recent study has reported cardiac damage after bouts of exercise in apparently healthy individuals [15] it is debated whether effect of exercise during physiological hypertrophy is truly a physiological incident that does not lead to pathological LV remodeling and cardiac dysfunction or long term chronic exercise training is maladaptive leading to compensated cardiac function and sudden death [5]. Researchers have pointed out increased cardiac risks in young athletes during or immediately after prolonged exercise training [5], [15]. However, the precise mechanism for such cardiac adaptation is not yet fully understood. Our study for the first time has identified specific isoforms of PKC associated to either physiological or pathological hypertrophy and also addressed the mechanism of such diverse cardiac adaptation by PKC-isoform switch.

## Materials and Methods

### Animal used

Balb/c mice (*Mus musculus*) used in this study were procured from National Institute of Nutrition, Hyderabad, AP, India. All experimental animals were maintained on standard mice chow and water ad libitum in a climate-controlled, light-regulated space with 12-hour light and dark cycles in the departmental animal facility of the University of Calcutta.

### Ethics statement

The investigation conforms to the Guidelines for the Care and Use of Laboratory Animals published by the US National Institute of Health (NIH Publication No. 85-23, revised 1996) and was also approved by the Institutional Animal Ethics Committee, University of Calcutta (Registration #885/ac/05/CPCSEA), registered under “Committee for the Purpose of Control and Supervision of Experiments on Laboratory Animals” (CPCSEA), Ministry of Environment and Forests, Government of India.

### *In vivo* generation of cardiac hypertrophy models

24 weeks old male Balb/c mice (n = 20) were used to generate various models for this study. Pathological cardiac hypertrophy was generated in a group of mice designated as H, by ligating right renal artery for 3 weeks described previously with some modifications [17]. Another group of mice designated as H*^X^*, were subjected to exercise training for the last 2 weeks of the 3 weeks tenure of renal artery ligation. Sham operated mice were used as controls.

Physiological cardiac hypertrophy was induced via swim exercise training for 3 weeks as described previously [18], [19] with some modifications (mice designated as E) whereas, another group designated as E*^R^*, was first subjected to exercise training for 3 weeks and maintained for 2 more weeks after exercise withdrawal. For time point specific studies, animals were rested for different time frames (viz., 3, 7, 15, 20, 30 and 45 days) after exercise withdrawal (n = 5). 24 week old male sedentary mice for similar period were used as control for this set. Each group of mice was sacrificed after the treatment period and the cardiac tissue was processed accordingly for various experimental purposes as described below.

### Isolation of cardiomyocytes from hearts of all experimental groups

Adult myocytes were isolated from hearts of all experimental mice groups following the procedure described previously [20]. ∼90% pure isolated cardiomyocytes were confirmed by staining with sarcomeric alpha-actinin antibody (Abcam, MA).

### Cardiac fibroblast culture and treatment

Fibroblast cells were isolated from 24 week old male mice hearts by the collagenase dispersion method [21]. 75–80% confluent and 24 h serum-starved cells were treated for 24 h with 10^−8^ mol/liter (Sar1)-Angiotensin-II (Bachem, CA). Ang-II-treated cells were used as positive controls for all subsequent experiments. Untreated cells were used as controls.

### *In vivo* treatment with chemical inhibitors and siRNA against PKC-δ and PKC-α

PKC-δ specific chemical inhibitor Rottlerin (Cat# R5648, Sigma-Aldrich, MO) and PKC-α specific chemical inhibitor Gö6976 (Cat# G1171, Sigma-Aldrich) [22] were dissolved in DMSO. Then along with 1X PBS, inhibitors were injected intraperitoneally in all three groups of experimental mice (C, H and E) at a dose of 600 µg/day/kg body weight during the last seven days of the experimental period as described earlier [23].

siRNAs against PKC-δ (siRNA ID: 151130; Catalogue no. # AM16708, Ambion, Life Technologies, NY) and PKC-α (siRNA ID: 151124; Catalogue no. # AM16708, Ambion, Life Technologies) as well as a nonspecific siRNA (Catalogue no. #4457289, Ambion, Life Technologies) at a concentration of 10 nmoles in 1X PBS was injected in ventricles in all three groups of experimental mice (C, H and E) following manufacturer’s protocol during the last seven days of the experimental period as described previously [24] with slight modification.

### Treatment of cardiac fibroblasts with PKC-δ inhibitor and siRNA

Rottlerin at a concentration of 3 µM and PKC-δ siRNA at a concentration of 10 nmoles were used in this study. Inhibitors were added 45 min before Ang-II treatment. Cells treated with equivalent concentration of DMSO and nonspecific siRNA (Catalogue no. #4457289, Ambion, Life Technologies, NY) were used as controls.

### Histology

All heart tissues were fixed in Karnovsky’s fixative, paraffin-embedded, and cut into 4 µm sections as described earlier [25]. Sections (taken from same areas of the heart of all the experimental animals) were stained with hematoxylin/eosin and all the stained sections were observed and captured under the microscope (BX-51, Olympus, PA) and myocyte dimensions were quantitated by a computer morphometric program (ImageJ, NIH). The cross-sectional areas were quantified in (>100) myocytes from each experimental group.

### Reverse transcriptase-PCR (RT-PCR)

Total RNA was isolated from all cardiac ventricular tissues using TRIzol reagent (Invitrogen, CA). Reverse transcription was done using Cloned AMV First-Strand cDNA Synthesis Kit (Invitrogen, CA) to check the expression of pathological hypertrophy marker genes, *Atrial natriuretic factor* (*ANF*) and *β Myosin heavy chain* (*β-MHC*) [17], [25] and *Insulin-like growth factor-1* (*IGF-1*) marker for physiological hypertrophy [15] using forward (F) and reverse (R) primers (IDT, San Diego, CA). *Glyceraldehyde-3-phosphate dehydrogenase* (*GAPDH*) was used as internal loading control.

[*ANF*-F5’-TGCCGGTAGAAGATGAGGTC-3′& R5’-AAGCTGTTGCAGCCTAGTCC-3′

*β-MHC*-F5’-CGGATGCCATACAGAGGAC-3′& R5’-CCTCATAGGCGTTCTTGAGC-3′

*IGF-1*-F5’-TGAGCTGGTGGATGCTCTCAGTT-3′& R5’-TCTGAGTCTGGGCATGTCATGT-3′

*GAPDH*-F5’-ACTCCACTCACGGCAAATTC-3′& R5’-TGTTGCTGTAGCCGTAT-3′]

### Protein extraction

After the experimental period, hearts were dissected out and perfused with chilled 1X phosphate buffered saline (PBS). Then the heart tissues were homogenized in protein extraction buffer [50 mM Tris, 250 mM NaCl 0.5% NP-40, 10% Glycerol, 5 mM EDTA, 0.1 M EGTA, 0.1 M PMSF, 1 M DTT, protease inhibitor (aprotinin, leupeptin and pepstatin) and phosphatase inhibitor cocktail (Sigma-Aldrich, MO)] and the supernatants were collected from each sample and concentrations of protein were estimated by Bradford assay at 595 nm using UV-vis Spectrophotometer (UV1700, Shimadzu Corporation, Kyoto, Japan) as described earlier [26].

Protein from adult cardiomyocytes was isolated using M-PER Mammalian protein extraction reagent (Thermo Scientific, IL). Mitochondrial and nuclear fraction from both cell and tissue samples were isolated by differential centrifugation method as described earlier [27], [28].

### Western blotting

30 µg of total protein and 200 µg of phospho protein samples were separated by SDS-PAGE and transferred to PVDF^+^ membrane (Millipore, MA), followed by incubation with primary antibodies against PKC-δ, phospho-PKC-δ (Thr 505), phospho-PKC-α (Ser 657 and Tyr 658), phospho-PKC-α/βII (Thr 638/641), PKC-α, PKC-βII, phospho-PKC-ε (Ser 729), PKC-ε, phospho-PKC-µ (Ser 744/748 and Ser 916), PKC-µ, phospho-PKC-δ/θ (Ser 643/676), phospho-PKC-θ (Thr 538), PKC-θ, phospho-PKC-ζ/λ (Thr 410/403), PKC-ζ, cytochrome-c, caspase-3, poly ADP ribose polymerase (PARP), phospho-P53 (Ser 46 and Ser 15), protein kinase-B (Akt), phospho-Akt, extracellular-signal-regulated kinase-1/2 (ERK-1/2), phospho-ERK1/2, STAT3, p38 MAPK, phospho-STAT3-Tyr-705, phospho-STAT3-Ser-727 and phospho-p38 MAPK (Cell Signaling, MA), bcl-2-associated X-protein (Bax) (BD Pharmingen, MD), P53, bH3-interacting domain death agonist (Bid), phospholipid scramblase-3 (PLS3) (Abcam, MA). 60S ribosomal protein L-32 (RPL32) (Abcam), Lamin-B and cytochrome-c Oxidase Subunit IV (COX IV) (Cell Signaling) were used as loading control for cytosolic proteins, nuclear proteins and mitochondrial proteins respectively. Immunoreactive bands were visualized using Immobilon Western chemiluminescence HRP substrate (Millipore, MA). The blots were scanned and quantitated using Gel Doc XR system and Quantity One software version 4.6.3 (Bio-Rad, CA).

### Total collagen estimation by hydroxyproline assay

Hydroxyproline assay was performed to measure ventricular collagen concentration both for *in vivo* experimental tissues as well as fibroblast culture supernatant (24 h treatment) [29]. Briefly, the tissue samples and fibroblast culture supernatants were subjected to acid digestion followed by vacuum drying. After resuspension in citrate acetate buffer, the samples were incubated with isopropyl alcohol, chloramine T, and Ehrlich’s reagent at 25°C for 18 h, and intensity of the red color was measured at 558 nm using Varioskan Multimode Reader (Thermo Fisher, IL). With the help of a standard curve, hydroxyproline content in the unknown samples was calculated. The amount of collagen was calculated by multiplying hydroxyproline content by a factor of 8.2.

### Caspase activity assay

Caspase activity was measured from all experimental cardiac tissues using ApoAlert caspase-3 and –9 Fluorescent Assay Kit (Clontech Laboratories, CA) following manufacturer’s protocol [17]. Briefly, tissue samples were homogenized in chilled protein extraction buffer. Then, 50 µl of 2X Reaction Buffer/DTT mix and 1 µl of Caspase-3 Inhibitor DEVD-CHO (for negative control) or 1 µl of DMSO (for other samples) was added to 50 µl of supernatant obtained from each sample. After incubation on ice for 30 min 5 µl of 1 mM Caspase-3 Substrate (DEVD-AFC; 50 µM final conc.) was added to each tube and incubated at 37°C for 1 hr. Fluorescence was measured at 400 nm excitation and 505 nm emission wavelengths (Varioskan Multimode Reader, Thermo Fisher, IL). For Caspase-9 activity, 5 µl of Caspase-9 Substrate (LEHD-AMC; 50 µM final conc.) was added to each tube and after incubation for 1 hr, fluorescence was measured at 380 nm excitation and 460 nm emission wavelength.

### Immunohistochemistry

Frozen ventricular tissue sections (4 µm) were prepared using cryostat CM1850 (Leica, CA) from all experimental groups. Tissue sections were fixed and stained with antibodies against phospho-PKC-δ and phospho-PKC-α (Cell signaling) and sarcomeric α-actinin (Abcam), followed by incubation with labeled secondary antibodies Alexafluor 488, and Alexa fluor 633 (Molecular Probes, OR) as described earlier [17]. After mounting with Vectashield [with DAPI] (Vector Laboratories, CA), tissue sections were visualized under confocal FV1200 microscope (Olympus, PA).

### Determination of cardiac function

Two-dimensional echocardiography was performed to determine cardiac function *in vivo* using ultrasound system (Vivid S5 system, GE Healthcare, WI) as described earlier [17]. M-mode views were used to assess the left ventricular chamber dimensions and functional parameters using digitized images captured during examination of each mouse.

### Statistical analysis

Results were expressed as mean ± S.E. of >3 independent experiments. Data was analyzed by independent samples t-test and ANOVA using SPSS (v13.0; IBM, NY). Values of P<0.05 were considered as significant.

## Results

### Assessment of cardiac hypertrophy in all the experimental groups

**Figure 1:**
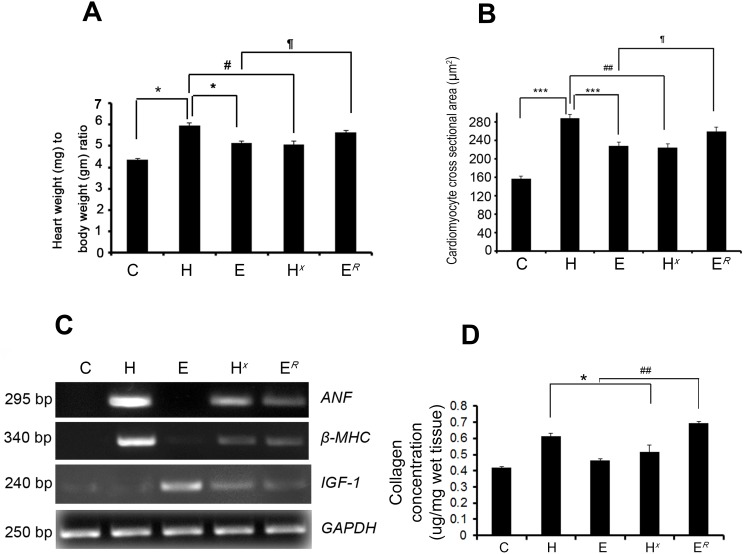
Assessment of hypertrophy and estimation of collagen in all the experimental groups. (**A**) Graph showing HW/BW ratio in all five experimental models: pathological hypertrophy (H), physiological hypertrophy (E), exercise-trained pathological hypertrophy (H*^X^*), mice kept at rest after 4 weeks of exercise training (E*^R^*) and representative control (C) (*p<0.05 for H versus E or C; #p<0.05 for H versus H*^X^*; ¶p<0.05 for E versus E*^R^*). (**B**) Graph showing cardiomyocyte cross-sectional area (in µm^2^) in groups C, H, E, H*^X^*, and E*^R^* (***p<0.001 for H versus E or C; ##p<0.01 for H versus H*^X^*; ¶p<0.05 for E versus E*^R^*). (**C**) Expression profile of pathological hypertrophy markers (*ANF* and *β-MHC*) and physiological hypertrophy marker (*IGF-1*) estimated by RT-PCR. *GAPDH* was used as loading control. (**D**) Graph showing ventricular collagen concentration in groups C, H, E, H*^X^*, and E*^R^* estimated by hydroxyproline assay.

Significant increase in the heart weight to body weight ratio (HW/BW) was observed in pathological as well as physiological hypertrophy (group H: 5.97±0.11 and group E: 5.12±0.12) compared to control group (4.34±0.06). Interestingly, the HW/BW ratio significantly increased in mice after exercise withdrawal (group E*^R^*: 5.65±0.09) compared to E and decreased in pathological hypertrophy group undergoing exercise training (group H*^X^*: 5.07±0.16; Figure 1A). Significant increase in expression of *ANF* and *β-MHC* in group E*^R^* (1.97±0.12-fold for *ANF* and 2.02±0.12-fold for *β-MHC*) compared to E, whereas, exercise training in pathological hypertrophy group showed significant down regulation in expression of both these hypertrophy marker genes (*ANF*: 2.44±0.03-fold and *β-MHC*: 2.14±0.02-fold in H*^X^* compared to H; Figure 1C, Figure S3). On the other hand, expression of *IGF-1*, the marker for physiological hypertrophy, that was increased significantly in group E (2.5±0.12-fold) compared to H or C, was drastically down regulated in group E*^R^* (1.78±0.02-fold) compared to E (Figure 1C, Figure S3). The cross-sectional area of cardiomyocytes was significantly increased in group H (288.62±7.75 µm^2^) and E (227.9±8.53 µm^2^) compared to C (157.45±4.99 µm^2^). However, the myocyte cross-sectional area was significantly increased in group E*^R^* (260.1±9.38 µm^2^) compared to E (Figure 1B). Similarly, ventricular collagen concentration was significantly increased in group H (0.613±0.02 µg/mg) compared to E and C. Interestingly, ventricular collagen concentration was significantly increased in mice after exercise withdrawal (group E*^R^*: 0.693±0.01 µg/mg) compared to E and was significantly decreased in pathological hypertrophy group undergoing exercise training (group H*^X^*) (0.517±0.04 µg/mg) compared to H (Figure 1D). Left ventricular chamber dimensions assessed by M-Mode echocardiography revealed significantly increased inter ventricular septum thickness (IVST) and posterior wall thickness (PWT) in group H (0.62±0.02 mm for IVST and 0.51±0.08 mm for PWT) and E (0.51±0.05 mm for IVST and 0.41±0.10 mm for PWT) compared to C (IVST 0.42±0.01 mm and PWT 0.26±0.06 mm) (Figure S1). Both IVST (0.47±0.01 mm) and PWT (0.31±0.04 mm) were significantly reduced in H*^X^* animals compared to group H indicating exercise after pathological hypertrophy ameliorates ventricular chamber thickness. Withdrawal from exercise in group E*^R^* revealed markedly increased IVST (0.59±0.02 mm), PWT (0.46±0.04 mm) (Figure S1) along with increased LVDD (3.07±0.05 mm) and decreased %FS (39±0.02%) compared to group E (LVDD 2.24±0.12 mm and 62.5±0.04%FS) manifesting severely compromised cardiac function in group E*^R^*, while cardiac function was markedly improved in group H*^X^* (LVDD 2.44±0.03 mm and %FS 55±0.33%) compared to group H (2.94±.05 mm for LVDD and 33±0.02% for %FS; Figure S2).

### Activation of PKC-isoforms during pathological and physiological hypertrophy

Phosphorylation status of different PKC-isoformswas estimated in C, H and E mice groups. Western blot analysis revealed no significant difference in phosphorylation levels of PKC-ε (Ser 729), PKC-α/βII (Thr 638/641), PKC-δ/θ (Ser 643/676), PKC-θ (Thr 538), PKC-ζ/λ (Thr 410/403), PKC-µ (Ser 744/748) and PKC-µ (Ser 916) in any of these groups (Figure S4 A). However, expression levels of total PKC-ε, PKC-ζ and PKC-θ increased significantly in group E and H compared to control, with no change in expression levels of total PKC-βII and PKD (Figure S4 A).

**Figure 2:**
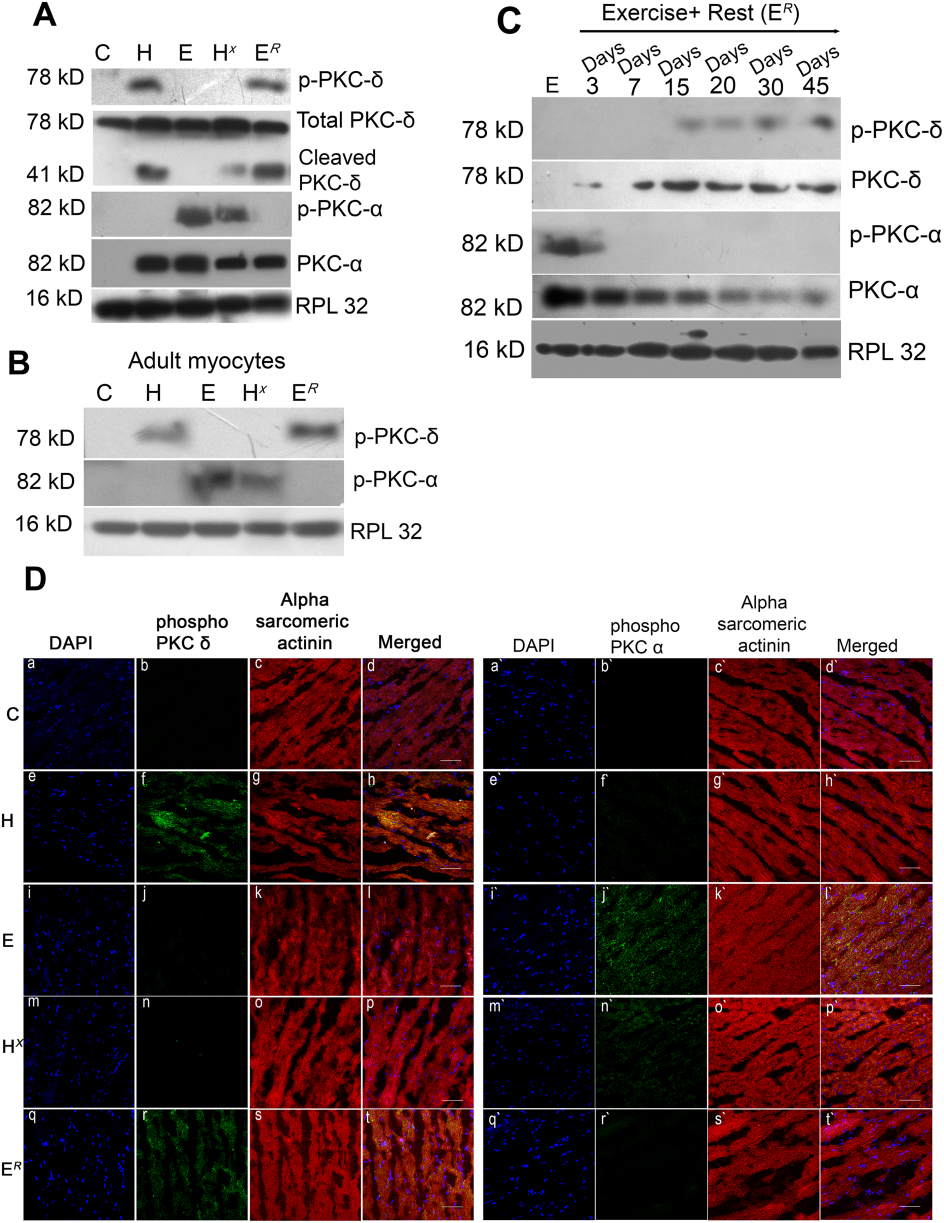
Differential expression profile of Protein Kinase-C isoforms. (**A**) Western blot analyses showing change in expression of PKC α and PKC-δ (phospho and total) in groups C, H, E, H*^X^* and E*^R^*. PKC-δ cleavage product (41 kD) is exclusive in group H, H*^X^* and E*^R^*. RPL32 was used as loading control. (**B**) Western blot analyses showing phosphorylation of PKC-δ and PKC-α in adult cardiomyocytes isolated from different experimental groups. RPL32 was used as loading control. (**C**) Western blot analyses showing changes in expression of phosphorylated and total PKC-δ and PKC-α in exercise withdrawn animals (E*^R^*) rested for different time periods. RPL32 was used as loading control. (**D**) Immunofluorescence study showing expression of phospho-PKC-δ and -α in different experimental group. Tissue sections showing phospho-PKC-δ expression in panels b, f, j, n and r and phospho-PKC-α in panels b’, f’, j’, n’ and r’ (green fluorescence). Sections were counter stained with alpha sarcomeric actinin antibody (panels c, g, k, o and s for PKC-δ and panels c’, g’, k’, o’ and s’ for PKC -α; red fluorescence). Nuclei were stained with DAPI (panels a, e, i, m and q for PKC-δ and panels a’, e’, i’, m’ and q’ for PKC-α; blue fluorescence) and merged images are shown in panels d, h, l, p and t for PKC-δ and panels d’, h’, l’, p’ and t’ for PKC-α. Increased expression of phospho-PKC-δ was observed in groups H and E*^R^* whereas, phospho-PKC-α expression was induced in group E and H*^X^* (Scale bar = 50 µm, Magnification = 40X).

However, phosphorylation level of PKC-δ (Thr 505) was found to be significantly high exclusively in group H (4.00±0.08-fold) compared to either C or E; whereas, significant increase in PKC-α phosphorylation level (Ser 657 and Tyr 658) was recorded in group E (6.13±0.02-fold) compared to either C or H (Figure 2A, 2B and S4 B). Interestingly, withdrawal from exercise in group E*^R^* showed significant down regulation in phospho-PKC-α level (4.73±0.07-fold) and induced phospho-PKC-δ level (2.75±0.11-fold) compared to group E, akin to what was observed in group H. On the contrary, significant increase in the levels of phospho-PKC-α (5.10±0.10-fold) and decrease in phospho-PKC-δ level (3.61±0.14-fold) was observed in group H*^X^* compared to H (Figure 2A, 2B and S4 B), similar to what is seen in group E. Total PKC-α expression increased in groups H (1.58±0.04-fold), E (1.30±0.04-fold) and H*^X^* compared to groups C and E*^R^* (Figure 2A). On the other hand, expression of total PKC-δ was increased in group H (4.97±0.07-fold) and E*^R^* (4.17±0.07-fold) compared to either C or E (Figure 2A). The western blot results were corroborated further by immunofluorescence studies that showed pronounced phosphorylation of PKC-δ in groups H and E*^R^* compared with either group C or E. On the other hand, phosphorylation of PKC-α was increased in groups E and H*^X^* compared to E, H or E*^R^* (Figure 2D).

Expression of PKC-δ and-α was studied in exercised withdrawn animals with increasing period of rest (3, 7, 15, 20, 30 and 45 days post exercise). Phosphorylation of PKC-δ was observed in exercise withdrawn animals from the 15^th^day of rest and was maximally induced on the 45^th^ day of rest period. Phosphorylation of PKC-δ was increased by 2.73±0.3-fold on the15^th^ day, 2.79±0.06-fold on the 20^th^ day, 2.88±0.15-fold on the 30^th^ day and 3.24±0.08-fold on the 45^th^ day of rest compared to exercised animals (E). However, phosphorylation of PKC-α was observed till the 3^rd^ day after exercise withdrawal but could not be detected with increasing time of rest (Figure 2C). Cardiac function was also found to deteriorate from 15^th^ day of rest and was almost equally compromised till 45^th^ day rest period as evidenced by significantly increased LVDD and reduced %FS, compared to 0 or 3 days of post exercise rest (Table 1).

**Table 1:** M-mode echocardiography data.

Experimental Groups	LVDD in mm	%FS	PW thickness in mm	IV septum thickness in mm
C	2.17±0.05	61±0.08%	0.26±0.06	0.42±0.01
H	2.94±0.05	33±0.02%	0.51±0.08	0.62±0.02
H*^X^*	2.44±0.03	55±0.02%	0.31±0.04	0.47±0.03
E	2.24±0.12	62.5±0.04%	0.37±0.01	0.5±0.05
E*^R 3days^*	2.30±0.15	57.5±1.32%	0.35±0.03	0.45±0.05
E*^R15days^*	3.07±0.05	39±0.03%	0.46±0.04	0.6±0.02
E*^R45days^*	2.99±0.06	37.5±2.33%	0.49±0.02	0.67±0.03

LVDD, left ventricular diastolic dimensions; FS, fractional shortening; PW thickness, posterior wall thickness; IV thickness, interventricular septum thickness.

### PKC-δ induces apoptotic signals in myocytes after exercise withdrawal during physiological hypertrophy

**Figure 3:**
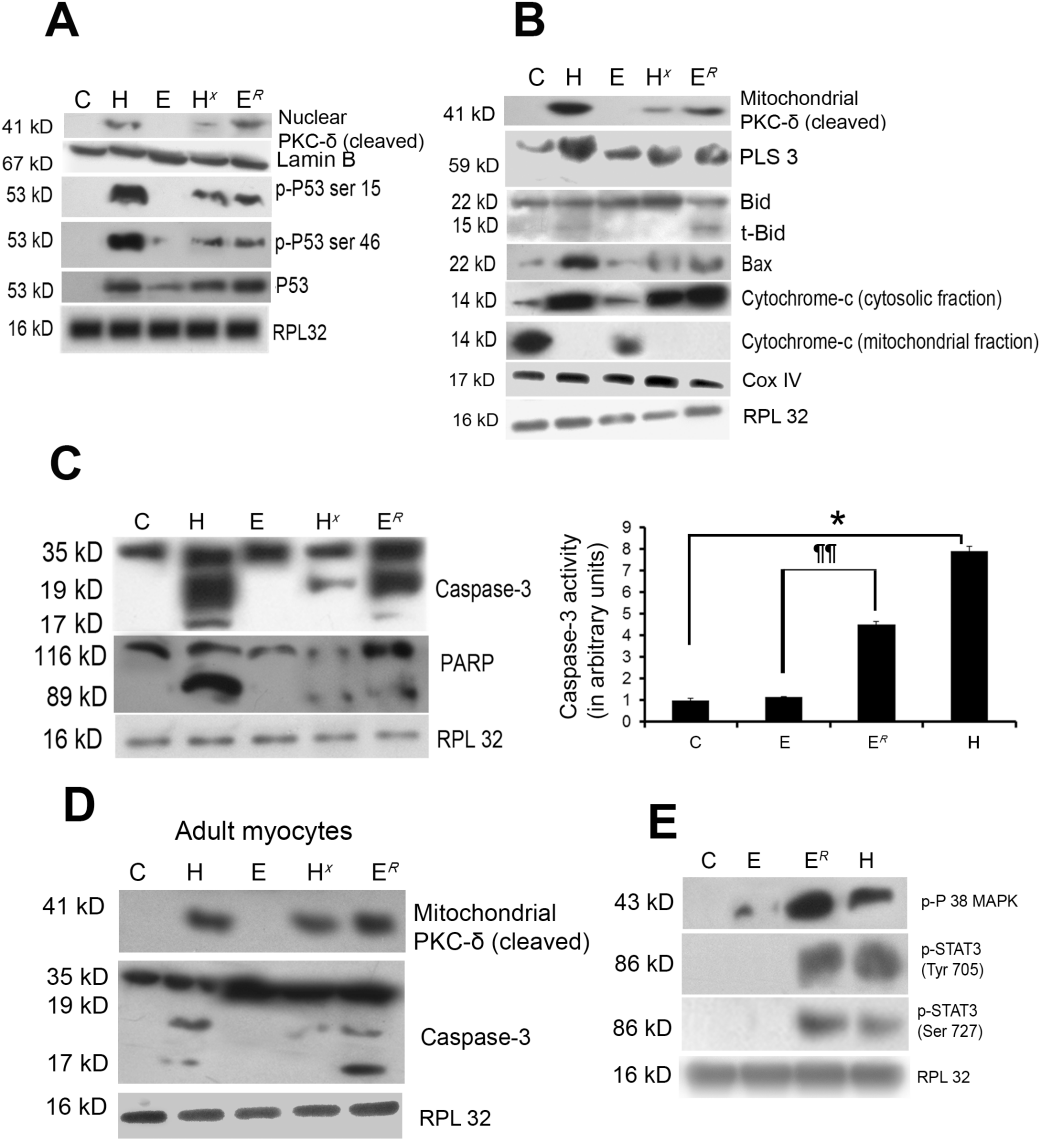
PKC-δ associated downstream target proteins. (**A**) Western blot analysis with the nuclear protein revealed significantly increased translocation of cleaved PKC-δ (41 kD) to nucleus in group H, H*^X^* and E*^R^* compared to either C or E. Significantly increased phospho-p53 (at Ser 46 and Ser 15) and total p53 was observed in group H and E*^R^* compared to E or C whereas significantly reduced phospho-p53 (at Ser 46 and Ser 15) and total p53 was observed in groups E and H*^X^* compared to H or E. RPL32 and Lamin B were used as loading control for cytosolic proteins and nuclear proteins respectively. (**B**) Subcellular fractionation followed by western blot analyses with mitochondrial protein showing significantly increased translocation of cleaved PKC-δ (41 kD) to mitochondria in group H, H*^X^* and E*^R^* compared to either C or E along with increased expression of PLS3, t-Bid, Bax, cytochrome-c proteins. RPL32 and COX IV were used as loading control for cytosolic proteins and mitochondrial proteins respectively. (**C**) Western blot analysis showingcleavage of caspase-3 and PARP in group H and E*^R^* compared to C or E. RPL32 was used as loading control. Caspase-3 activity assay showing similar changes in all the experimental groups. No significant difference in caspase-3 activity was detected between groups E and C (*p<0.05 for H versus C; ###p<0.001 for H versus H*^X^*; ¶¶p<0.01 for E versus E*^R^*). (**D**) Subcellular fractionation followed by western blot analyses showed significantly increased translocation of cleaved PKC-δ (41 kD) to mitochondria along with caspase-3 cleavage in adult cardiomyocytes isolated from group H and E*^R^* compared to either C or E or H*^X^*. RPL32 was used as loading control. (**E**) Western blot analysis showing phosphorylation status of STAT3 and P38 MAPK in groups C, E, E*^R^* and H.

Subcellular fractionation followed by western blot analysis in different experimental groups revealed cleavage of PKC-δ and translocation of its active subunit (41 kD) to mitochondria and nucleus in group H and to a lesser extent in group H*^X^* (Figure 3A). No translocation of PKC-δ active subunit was observed in groups C or E. However, when exercise was withdrawn in physiological hypertrophy (group E*^R^*), nuclear translocation was observed (Figure 3A and 3B). Similar translocation pattern of active PKC-δ fragment was observed in isolated adult cardiomyocytes from animals of all experimental groups (Figure 3D).

Expression level of total and phosphorylated p53 (ser-46, ser-15) was observed in group H compared to groups C and E. However, phosphorylated p53 band reappeared in group E*^R^* (4.43±0.12-fold for total p53, 4.66±0.20-fold at ser-46 and 3.56±0.2-fold at ser-15 compared to E; Figure 3A) and phosphorylation level in group H*^X^* was significantly reduced compared to H (Figure 3A).

The expression of proapoptotic proteins downstream to PKC-δ, such as phospholipid scramblase-3 (PLS3), bcl-2 associated X-protein (Bax), cleavage of bH3-interacting domain death agonist (Bid) to truncated-Bid (t-Bid) and cytosolic/mitochondrial ratio of cytochrome-c were significantly increased in group E*^R^* (1.91±0.02-foldfor PLS3, 2.83±0.08-fold for Bax and 11.09±0.13-fold for cytochrome-c) as was induced in group H compared to either C or E (Figure 3B).

Western blot analysis with whole heart and isolated cardiomyocytes from different experimental groups revealed active catalytic fragments (19 kD and 17 kD) of cleaved caspase-3 during exercise withdrawal group E*^R^* that were absent in group E (Figure 3C and 3D). Caspase-3 activity assay showed similar trend with significantly induced activity in group H and E*^R^* (3.28±0.69-fold) compared to E (Figure 3C). Appearance of cleaved 89 kD active fragment of Poly ADP ribose polymerase (PARP) which is a hallmark of pathological hypertrophy (H) was also detected in group E*^R^* (Figure 3D). In tune with this, significant increase in phosphorylation of STAT3 (at Tyr-705 and Ser-727) and P38 MAPK was observed in group E*^R^* compared to groups E or C (Figure 3E).

**Figure 4:**
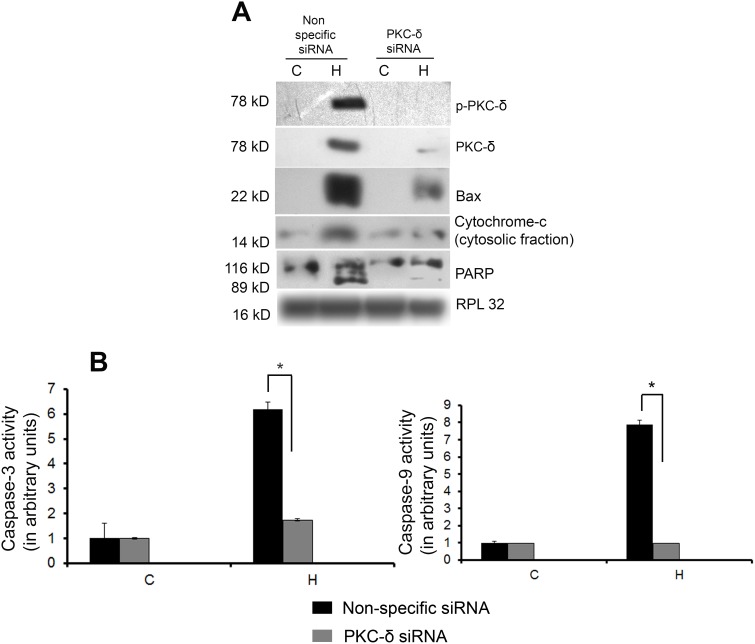
Inhibition of PKC-δ reduces expression of downstream targets. (**A**) Western blot analysis showing successful down regulation in expression of both phospho and total PKC-δ along with significant decrease in the level of bax, cytosolic cytochrome-c and PARP cleavage in mice treated with PKC-δ siRNA in group H. RPL32 was used as loading control. (**B**) Graph showing caspase-3 and caspase-9 activities in the two experimental groups (C and H) treated with siRNA and nonspecific siRNA. Significant decrease in caspase-3 and caspase-9 activities occurred in mice treated with PKC-δ siRNA compared to mice treated with nonspecific siRNA [*p<0.05 for H (nonspecific siRNA) versus H (PKC-δ siRNA)].

Both phosphorylated and total PKC-δ expression was significantly reduced in PKC-δ siRNA treated pathological hypertrophy group (H) along with significant down regulation of Bax (2.91±0.06-fold), cytosolic/mitochondrial ratio of cytochrome-c (6.29±0.08-fold) and cleavage of PARP compared to nonspecific siRNA treated group H (Figure 4A and S5). The PKC-δ siRNA treatment also resulted in significant reduction in activities of caspase-3 and -9in group H (7.83±0.53 and 3.56±0.28-fold respectively) compared to nonspecific siRNA treated group H mice (Figure 4B). Similar results were observed when group H mice were treated with chemical inhibitor Rottlerin compared to vehicle treated ones (data not shown). PKC-δ siRNA or Rottlerin treatment did not alter the expression of these proteins during physiological hypertrophy (data not shown).

### Down regulation of PKC-α promotes deactivation of Akt and ERK-1/2 mediated cell survival pathway during exercise withdrawal

**Figure 5:**
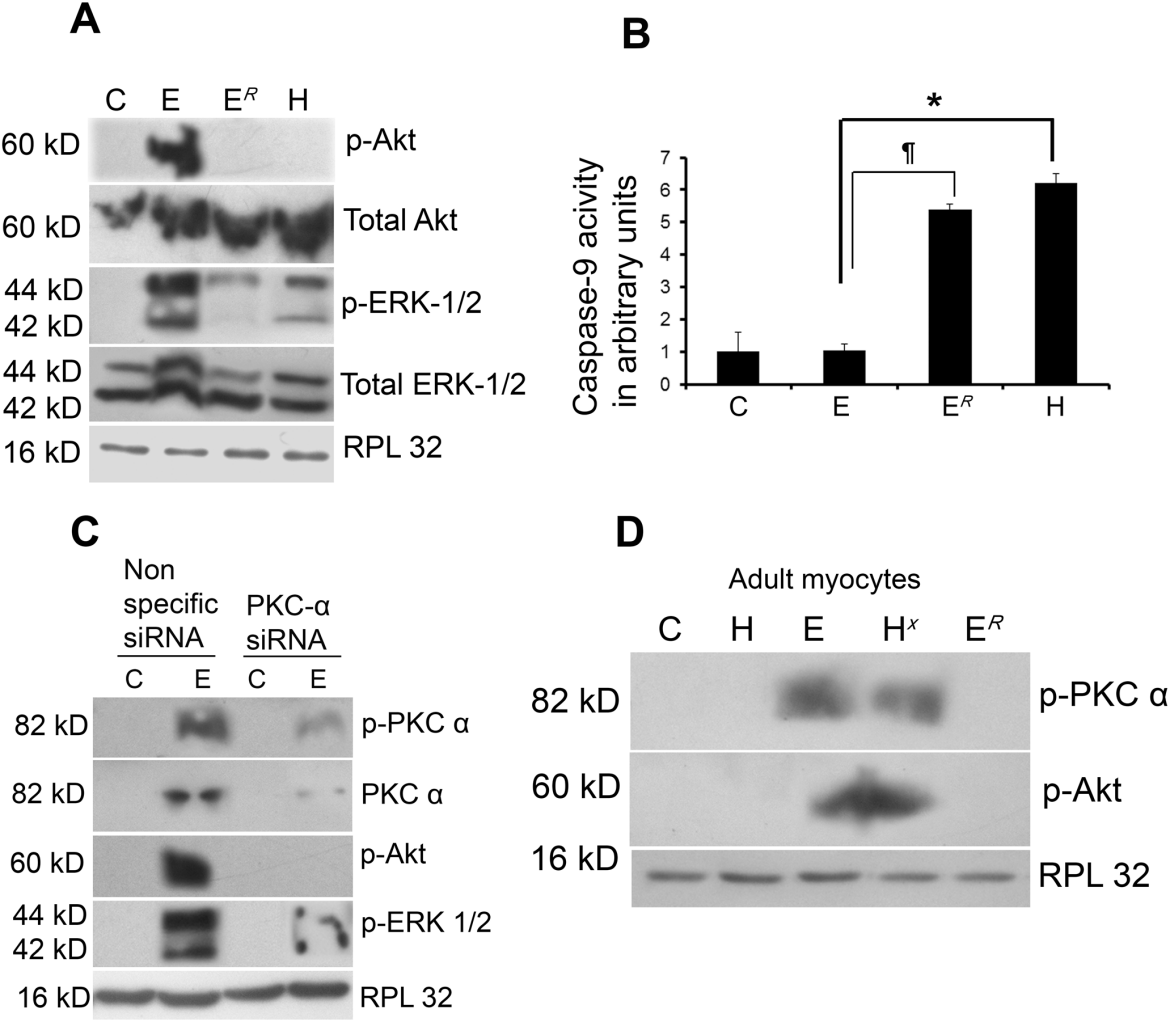
PKC-α and survival kinases. (**A**) Western blot analysis showing phospho-Akt to Akt and phospho-ERK-1/2 to ERK-1/2 ratio to be significantly increased in group E compared to either H or C. phospho-Akt/Akt and phospho-ERK-1/2 to ERK-1/2 ratio was significantly decreased in group E*^R^* compared to E. RPL32 was used as loading control. (**B**) Graph showing caspase-9 activity in groups C, E, E*^R^* and H. (*p<0.05 for H versus E; ¶p<0.05 for E versus E*^R^*). (**C**) Western blot analyses reveal successful knockdown of phospho and total PKC-α along with significant decrease in the phosphorylation level of Akt and ERK-1/2 in mice treated with PKC-α siRNA. RPL32 was used as loading control. (**D**) Phosphorylation of PKC-α and Akt was observed by western blot analysis in adult cardiomyocytes isolated from group E and H*^X^* compared to C, H and E*^R^*. RPL32 was used as loading control.

The phosphorylation status of Akt and ERK-1/2, downstream pro-survival targets of PKC-α, was studied in all the experimental groups. Western blot analyses revealed phosphorylation of Akt (serine 473) only in group E and phospho-Akt to Akt ratio was decreased by 1.52±0.02-fold during exercise withdrawal in group E*^R^* (Figure 5A and S6) whereas, exercise regimen during pathological hypertrophy (H*^X^*) resulted in Akt phosphorylation that was absent in group H (data not shown). Similarly, phospho-ERK-1/2 to ERK-1/2 ratio was increased significantly in group E (5.3±0.02-fold) compared to C but decreased in group E*^R^* (1.44±0.15-fold) compared to E (Figure 5A and S6). Activity assay of proapoptotic caspase-9 protein revealed significant down regulation in group E compared to H (5.95±0.67-fold). Interestingly, caspase-9 activity was found to increase significantly in group E*^R^* (5.17±0.50-fold) compared to E (Figure 5B). Cardiomyocytes isolated from experimental groups showed similar trend in phosphorylation level of PKC-α and Akt (Figure 5D).

Pro-survival role of PKC-α during physiological hypertrophy was further confirmed by treating exercised mice group (E) with PKC-α siRNA. Significant down regulation in the levels of phospho and total PKC-α along with phospho-Akt and phospho-ERK-1/2 levels was observed in group E mice treated with PKC-α siRNA compared to nonspecific siRNA treatment (Figure 5C and S7). G6976 (chemical inhibitor of PKC-α) treatment showed similar results for phosphorylation of Akt and ERK levels (data not shown). Inhibition of PKC-α activity did not alter phosphorylation status of either Akt or ERK-1/2 during pathological hypertrophy (H) (data not shown).

### Inhibition of PKC isoforms reverses cardiac adaptation during pathological and physiological hypertrophy

#### PKC-α inhibition activates PKC-δ during physiological hypertrophy

**Figure 6:**
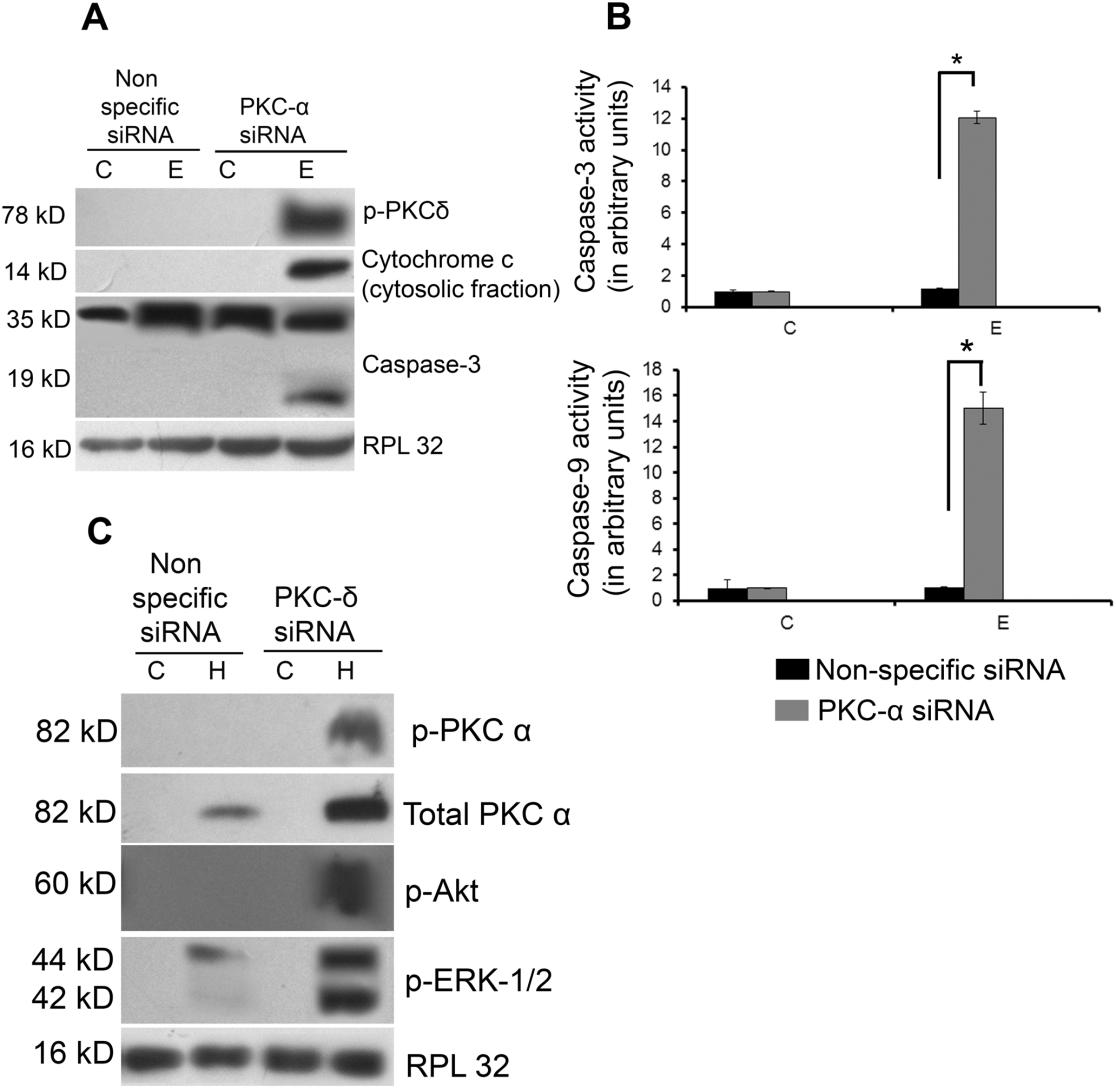
Reversal of PKC isoforms modulates hypertrophy regulators. (**A**) Western blot analysis showing inhibition of PKC-α in physiological hypertrophy mice with PKC-α siRNA resulting in significant phosphorylation of PKC-δ and translocation of active PKC-δ (41 kD), cytosolic cytochrome-c and caspase-3 cleavage compared to nonspecific siRNA treated physiological hypertrophy mice. RPL32 was used as loading control. (**B**) Graph showing PKC-α siRNA treatment during physiological hypertrophy generation showed significant increase in caspase-3 and caspase-9 activities compared to nonspecific siRNA treatment during physiological hypertrophy [*p<0.05 for E (nonspecific siRNA) versus E (PKC-α siRNA)]. (**C**) Western blot analyses showing significantly increased phosphorylation of PKC-α, Akt and ERK-1/2 and PKC-α expression in pathological hypertrophy mice treated with PKC-δ siRNA. RPL32 was used as loading control.

PKC-α siRNA treatment during physiological hypertrophy led to significant increase in the phospho-PKC-δ level in group E mice along with increase in ratio of cytosolic/mitochondrial cytochrome-c (8.74±0.2-fold, p<0.01) and cleavage of caspase-3 compared to nonspecific siRNA treated group E mice (Figure 6A). Significantly induced caspase-3 (10.54±0.06-fold, p<0.05) and caspase-9 (14.42±2.16-fold, p<0.05) activities were also recorded in PKC-α siRNA treated group E samples (Figure 6B). However, inhibition of PKC-α during pathological hypertrophy did not show alteration of phospho-PKC-δ expression and apoptotic markers in group H (data not shown). Apart from induced apoptotic signaling, altered activation from PKC-α to PKC-δ resulted in compromised cardiac function with significantly increased LVDD and decrease in %FS in PKC-α siRNA treated group E mice, compared to nonspecific siRNA treated mice of the same group (LVDD: 2.87±0.02 mm vs 2.24±0.12 mm; %FS: 49.8±0.02% vs 62.5±0.04%; p<0.05) (Figure S8).

#### Inhibition of PKC-δ activates PKC-α isoform and subsequent Akt-ERK 1/2 activation during pathological hypertrophy

PKC-δ siRNA treated group H mice on the other hand, showed significant increase in the level of both phospho-PKC-α (5.73±0.33-fold, p<0.01) and total PKC-α (1.54±0.04-fold, p<0.01) level compared to nonspecific siRNA treated pathological hypertrophy group (Figure 6C). Inhibition of PKC-δ also significantly increased the expression of phospho-Akt (2.57±0.04-fold, p<0.01) and phospho-ERK-1/2 (2.61±0.02-fold, p<0.01) in this mice group (Figure 6C). However, PKC-δ inhibition did not alter expression of phospho-Akt, phospho-ERK-1/2 in group E (data not shown). Inhibition of PKC-δ and subsequent activation of phopho-PKC-α level in group H resulted in notably improved cardiac function with significant decrease in LVDD (1.75±0.05 mm, p<0.05) and increase in %FS (72.6±0.03%, p<0.05) compared to otherwise severely compromised functional parameters in nonspecific siRNA treated mice with pathlogical hypertrophy (Figure S9).

### Inhibition of PKC-δ modulates collagen concentration via STAT3 and P38 MAPK

**Figure 7:**
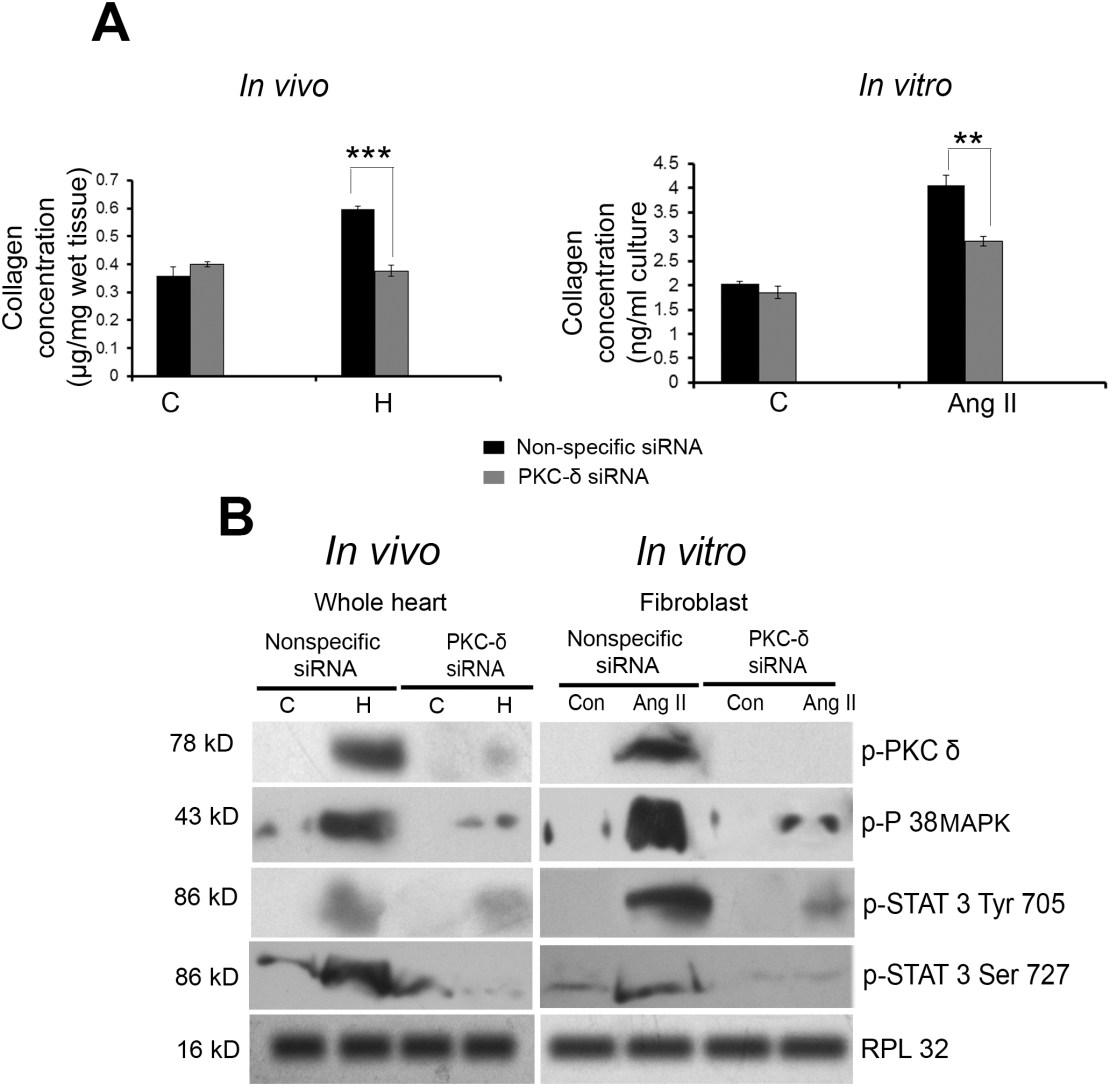
Effect of PKC-δ down regulation on collagen synthesis during pathological hypertrophy. (**A**) Graph showing ventricular collagen concentration in groups C, H and H + PKC-δ siRNA (*in vivo*) and in groups C, Ang-II and Ang-II + PKC-δ siRNA (*in vitro*) estimated by hydroxyproline assay. [***p<0.01 for H (nonspecific siRNA) versus H (PKC-δ siRNA) and **p<0.01 for Ang-II (nonspecific siRNA) versus Ang-II (PKC-δ siRNA)]. (**B**) Western blot analysis showing status of phospho PKC-δ, phospho P38, phospho STAT3 (Tyr 705 and Ser 727), STAT3 following PKC-δ siRNA treatment along with pathological hypertrophy (H) in comparison to hypertrophy alone *in vivo* as well as *in vitro*. RPL32 was used as loading control.

Inhibition of PKC-δ resulted in significant regression of ventricular collagen concentration in siRNA treated pathological hypertrophy (H) (0.377±0.02 µg/mg) samples as well as in Ang-II treated cardiac fibroblast (2.90±0.01 ng/ml) compared to nonspecific siRNA treated group H (0.597±0.01 µg/mg) and cardiac fibroblast (4.06±0.21 ng/ml) respectively (Figure 7A).

PKC-δ siRNA treated pathological hypertrophy mice group (H) and Ang-II treated cardiac fibroblast showed significantly reduced phosphorylation status of PKC-δ along with P38 MAPK. The inhibitor treatment also resulted in reduction of phosphorylation of STAT3 (Tyr-705 and Ser-727) in group H mice as well as in Ang-II treated cardiac fibroblast compared to nonspecific siRNA treated group H and cardiac fibroblast (Figure 7B). *In vivo* and *in vitro* treatment with PKC-δ siRNA had no significant effect on total P38 and STAT3 (data not shown). Similar reduction in expression of these proteins was observed following Rottlerin treatment in group H mice and Ang-II treated cardiac fibroblast (data not shown).

## Discussion

PKC has been identified as an important player during cardiac hypertrophy [6]. However, isoform specific roles of PKC during pathological and physiological hypertrophy have never been reported. This study for the first time revealed how differential activation and switch of PKC-isoforms dictate cardiomyocyte adaptation via key downstream modulators to regulate cardiac efficiency during physiological and pathological hypertrophy.

Pathological hypertrophy in group H was confirmed by significant increase in HW/BW ratio, induced expression of hypertrophy markers *ANF* and *β-MHC* along with ventricular collagen concentration, a marker of cardiac fibrosis [17], [25]. Physiological hypertrophy by exercise training was characterized by increase in HW/BW ratio and *IGF-1* expression in group E [15]. Cardiomyocyte cross-sectional areas were increased significantly in both groups H and E compared to C (Figure 1A, 1C, 1D and S3). Left ventricular chamber dimensions showed significant thickening during pathological hypertrophy (Group H) as evidenced by increased IVST, PWT, LVDD (Figure S1 and S2) and decreased %FS (Figure S2) confirming severely compromised cardiac function. Physiological hypertrophy (Group E) was marked by increased LVDD but no significant change in %FS with that of control animals confirming efficient cardiac contractibility. Interestingly, when group E mice were withdrawn from the exercise regimen for two weeks (group E*^R^*) significant up regulation of *ANF* and *β-MHC* and severely compromised cardiac function was observed indicating development of pathological hypertrophy, akin to group H (Figure 1A, 1C, 1D and S1, S2, S3).

Several studies had already indicated a prominent role of PKC during cardiac hypertrophy [7]–[12] but the precise role of specific PKC-isoforms encompassing various forms of hypertrophy processes is still not understood. Our study revealed altered expression and differential phosphorylation of PKC-α and -δ in group E and H respectively, while activation of other PKC-isoforms remained more or less unaltered among different mice groups (Figure 2A and S4 A-S4 B). Phosphorylation of PKC-δ at Thr 505 was detected exclusively in group H while phosphorylated PKC-α at Ser 657 and Tyr 658 was observed in group E, suggesting their exclusive activation during pathological and physiological hypertrophy respectively (Figure 2A–2B, 2D and S4 B). On the contrary, withdrawal from exercise regimen in group E*^R^* resulted in reduction in phospho-PKC-α level compared to E with significant increase in total and phospho-PKC-δ whereas, significant increase in phospho-PKC-α and reduced phospho-PKC-δ level were the hallmarks of group H*^X^* (Figure 2A–2B, 2D and S4 B). This is the first report where involvement of specific PKC-isoforms -δ and -α was recorded in pathological and physiological hypertrophy respectively with strategic reversal of the same during reversed hypertrophic conditions evidenced by PKC-δ activation in E*^R^* and PKC-α in H*^X^* groups suggesting that transition from PKC-α to -δ activation could dictate alteration from adaptive to compromised cardiac hypertrophic condition in animal model. Time point study with E*^R^* group of mice showed increasing trend of phosphorylation of PKC-δ from 15^th^day of rest which increased progressively till the 45^th^day (Figure 2C), with progressive deterioration in cardiac performance (Table 1). Phosphorylation of PKC-α, on the other hand, started declining progressively from the 3^rd^day onwards after exercise withdrawal. This was an interesting observation as our data suggests that post exercise rest period could lead towards negative cardiac remodeling in hearts with efficient performances during physiological hypertrophy.

Study of downstream signaling pathways regulated by the specific PKC-isoforms during pathological and physiological hypertrophy revealed that induced PKC-δ phosphorylation and its increased translocation to mitochondria and nucleus during pathological hypertrophy (Figure 3A, 3B and 3D) could induce cardiomyocyte apoptosis in group H, as reported earlier where overexpression of PKC-δ catalytic fragment and its translocation to subcellular organelles induced apoptosis in various cell types and overexpression of PKC-δ kinase ‘dead mutants’ protected cells from apoptosis [30]. Translocated active PKC-δ interacts with PLS3 [31] that increases cardiolipin expression on the outer mitochondrial membrane [32] thereby recruiting.

t-Bid for eventual cytochrome-c release via formation of Bax/Bak pores [33]. Significant increase in activation and translocation of PLS3, cleavage of Bid to t-Bid, Bax, cytosolic cytochrome-c, phospho-P53 level [30] along with induced cleavage of caspase-3, an upstream activator of PKC-δ [34] and PARP, a known substrate of caspase-3 [28] was observed in group H compared to other groups (Figure 3A–3D). Proapoptotic role of PKC-δ during pathological hypertrophy was further confirmed by inhibiting both phosphorylated and total PKC-δ activity in group H mice that showed significant reduction in the levels of apoptotic regulators (Figure 4A, 4B and S5). In tune with our earlier results all these markers were increased significantly in E*^R^* after exercise withdrawal in physiological hypertrophy group (Figure 3A–3D). Thus, it can be inferred that PKC-δ activation during pathological hypertrophy induces cardiomyocyte apoptotic load resulting in compromised cardiac function [17], [28] similar to what was recorded in E*^R^* mice that has been withdrawn from a continuous exercise training regimen during physiological hypertrophy. Our results also suggest that exercise training could play a crucial role in the deactivation of proapoptotic PKC-δ and its downstream modulators resulting in improved cardiac efficiency during pathological hypertrophy in group H*^X^* (Figure 3A–3D).

Activation of caspase-3 via the intrinsic pathway is a critical mediator of the hypertrophic responses [28], [35]. In addition to regulation of hypertrophic responses, caspase-3 has been shown to cleave PKC-δ for its activation during apoptosis in vascular smooth muscle cells as well as in a wide range of other cell types [30], [34]. Our data has shown translocations of cleaved PKC-δ into mitochondria and nucleus (Figure 3A–3B) which in turn induces caspase-9 mediated caspase-3 cleavage thus forming a feedback loop that further exacerbates the hypertrophic responses [30]. These results cumulatively demonstrate that caspase mediated signaling is a key driver of the pathologic hypertrophy state and establish a clear link between caspase-3 signaling and cleavage activation of various PKC isoforms [36], [37].

PKC-α on the other hand, has been reported by many to be a pro-survival isoform that promotes cell survival via direct phosphorylation of Akt at serine 473 [38] and also as a negative regulator of cardiac functional efficiency shown by its increased expression in various models of cardiac injury or failure [12], [39]. PKC-α dependent regulation of cardiomyocyte hypertrophy requires ERK-1/2 activation [9] for cardio-protection during ischemia reperfusion injury through activation of the several anti-apoptotic and pro-survival signaling cascades [40]. Our study has revealed significantly increased phosphorylation of Akt and ERK-1/2 along with exclusive activation of PKC-α in group E during physiological hypertrophy (Figure 5A and S6). Their pro-survival role in these animals was also ascertained by significantly decreased caspase-9 activity (Figure 5B), corroborating earlier reports that claimed responsibility of these kinases for the inactivation of proapoptotic caspase-9 [41]. Inhibition of both phosphorylated and total PKC-α during physiological hypertrophy resulting in significant deactivation of phospho-ERK-1/2 and -Akt level further confirms the pro-survival role of PKC-α (Figure 5C and S7) and indicates that exercise training could play a crucial role in the activation of PKC-α and its downstream pro-survival proteins. In contrast, deactivation of PKC-α along with decreased phosphorylation of Akt and ERK-1/2 leading to the activation of pro-apoptotic caspase-9 recorded in group E*^R^* (Figure 5A–5B and 5D) again confirms that withdrawal from exercise training during physiological hypertrophy leads to onset of pathological hypertrophic characters marked by induction of myocyte apoptosis and compromised cardiac function in group E*^R^*. Moreover PKC-δ inhibition in group H interestingly, led to the reactivation of PKC-α along with induced downstream pro-survival markers viz. phospho-Akt and phospho-ERK-1/2 with significant improvement of cardiac function during pathological hypertrophy (Figure 6C and S9) similar to the condition achieved through exercise training during pathological hypertrophy in group H*^X^*. This result was corroborated by an earlier report where PKC-δ inhibition led to activation of PKC-α isoform in prostate cancer cells [42]. Simultaneously, PKC-δ inhibition also resulted in significant decrease in ventricular collagen concentration in both *in vivo* and *in vitro* models of pathological hypertrophy along with significant reduction of phospho-STAT3 (at Tyr-705 and Ser-727; Figure 7A and 7B), an important regulator of ventricular collagen synthesis as described earlier by our group [25].

On the other hand, PKC-α inhibition during physiological hypertrophy resulted in activation of PKC-δ in group E along with induction of apoptotic regulator proteins and severely compromised cardiac function (Figure 6A–6B and S8) similar to the phenotypes observed in group E*^R^.* This result corroborates with an earlier report that showed induced PKC-δ dependent apoptotic program upon inhibition of PKC-α in salivary epithelial cells [14].

Thus, our study clearly demonstrated that PKC-α and -δ isoforms are the prime modulators of cardiac adaptation during physiological and pathological hypertrophy respectively. Further, this study also established that cardiac adaptive processes during transition of an efficiently functioning heart to a functionally compromised heart could be modulated by reversal of activation of PKC-α to-δ isoform.

## Supporting Information

Figure S1 **M-mode echocardiography parameters.** Graph showing IVST and PWT in all experimental groups (*p<0.05 for H versus E or C; #p<0.05 for H versus H*^X^*; ¶p<0.05 for E versus E*^R^*).
(TIF)

Figure S2 **M-mode echocardiography parameters.** Graph showing LVDD and %FS in all experimental groups (*p<0.05 for H versus E or C; #p<0.05 for H versus H*^X^*; ¶p<0.05 for E versus E*^R^*).
(TIF)

Figure S3 **Band intensities of hypertrophy markers.** Graph showing pathological hypertrophy markers (*ANF* and *β-MHC*) and physiological hypertrophy marker (*IGF-1*) estimated by RT-PCR. *GAPDH* was used as loading control. (For *ANF* ***p<0.001 for E versus E*^R^*; ###p<0.001 for H versus H*^X^*; For *β-MHC* ****p<0.001 for E versus E*^R^*; ###p<0.001 for H versus H*^X^*; For *IGF-1* ***p<0.001 for E versus H; ###p<0.001 for E versus E*^R^*).
(TIF)

Figure S4 **Expression level of different PKC-isoforms. (A)** Western blot analyses showing status of phospho-PKC-isoforms [PKC-ε (Ser 729), PKC-α/βII (Thr 638/641), PKC-δ/θ (Ser 643/676), PKC-θ (Thr 538), PKC-ζ/λ (Thr 410/403), PKC-µ (Ser 744/748) and PKC-µ (Ser 916)] and total PKC-isoforms (PKC-ε, PKC-ζ, PKC-βII and PKD) in group C, H and E. RPL32 was used as loading control. **(B)** Graph showing relative band intensity of phospho-PKC-δ and phospho-PKC-α in C, H, E, H*^X^* and E*^R^* as revealed by western blot analysis (For phospho-PKC-δ: ***p<0.001 for H versus C or E; ###p<0.001 for H versus H*^X^*; ¶¶p<0.01 for E versus E*^R^*. For phospho-PKC-α: **p<0.01 for E versus H or C; ###p<0.001 for H versus H*^X^*; ¶¶p<0.01 for E versus E*^R^*).
(TIF)

Figure S5 **Silencing of PKC-δ results in down regulation of apoptotic markers.** Graph showing significantly decreased phospho-PKC-δ, Bax and cytochrome-c (cytosolic/mitochondrial ratio) in mice treated with PKC-δ siRNA compared to nonspecific siRNA treated mice [*p<0.05 for H (nonspecific siRNA) versus H (PKC-δ siRNA)].
(TIF)

Figure S6 **Relative expression of Akt and ERK-1/2.** Graph showing phospho-Akt/Akt and phospho-ERK-1/2/ERK-1/2 ratio in group C, E, E*^R^* and H (For phospho-Akt/Akt ratio: ***p<0.001 for E versus C; ¶¶p<0.01 for E versus E*^R^*. For phospho-ERK-1/2/ERK-1/2 ratio: ***p<0.001 for E versus C; ¶¶p<0.01 for E versus E*^R^*.
(TIF)

Figure S7 **Silencing of PKC-α results in down regulation of prosurvival markers.** Graph showing significantly decreased phospho-PKC-α along with significant decrease in the phosphorylation level of Akt and ERK-1/2 in mice treated with PKC-α siRNA compared with nonspecific siRNA treated mice [*p<0.05 for E (nonspecific siRNA) versus E (PKC-α siRNA)].
(TIF)

Figure S8 **Cardiac functional parameters after PKC-α silencing.** Graph showing significant increase in LVDD and decrease in %FS in mice treated with PKC-α siRNA compared to nonspecific siRNA treated mice [*p<0.05 for E (nonspecific siRNA) versus E (PKC-α siRNA)].
(TIF)

Figure S9 **Cardiac functional parameters after PKC-δ silencing.** Graph showing significant decrease in LVDD and increase in %FS in mice treated with PKC-δ siRNA compared to nonspecific siRNA treated mice [*p<0.05 for H (nonspecific siRNA) versus H (PKC-δ siRNA)].
(TIF)

Raw Data S1 (RAR)
