# Supplementary figures and images for: Differential and Conditional Activation of PKC-Isoforms Dictates Cardiac Adaptation during Physiological to Pathological Hypertrophy
Source: PLoS One. 2014 Aug 12;9(8):e104711. doi: 10.1371/journal.pone.0104711 (PMC4130596; doi:10.1371/journal.pone.0104711)

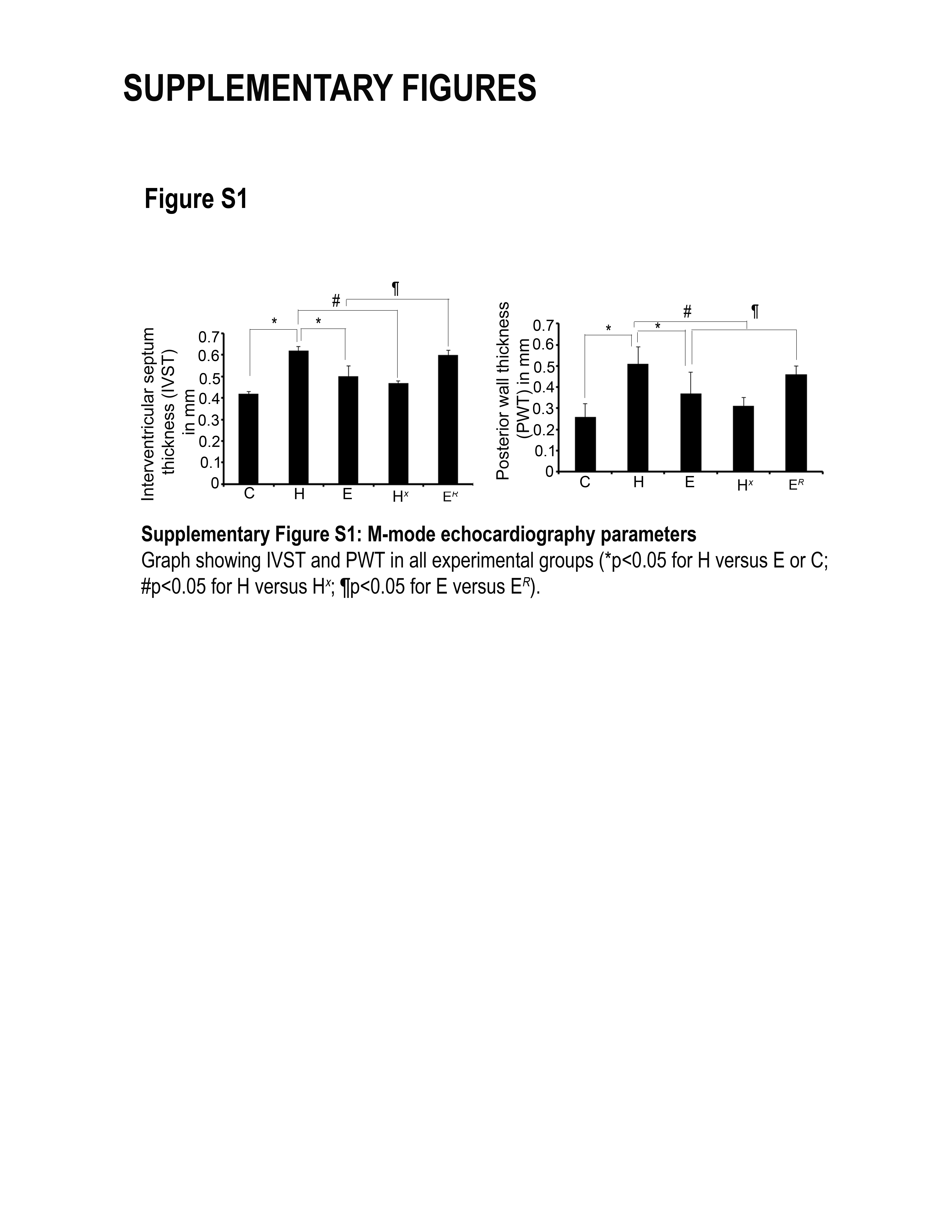

Supplement: Figure S1 — M-mode echocardiography parameters. Graph showing IVST and PWT in all experimental groups (*p<0.05 for H versus E or C; #p<0.05 for H versus HX; ¶p<0.05 for E versus ER). (TIF) [file pone.0104711.s001.tif]

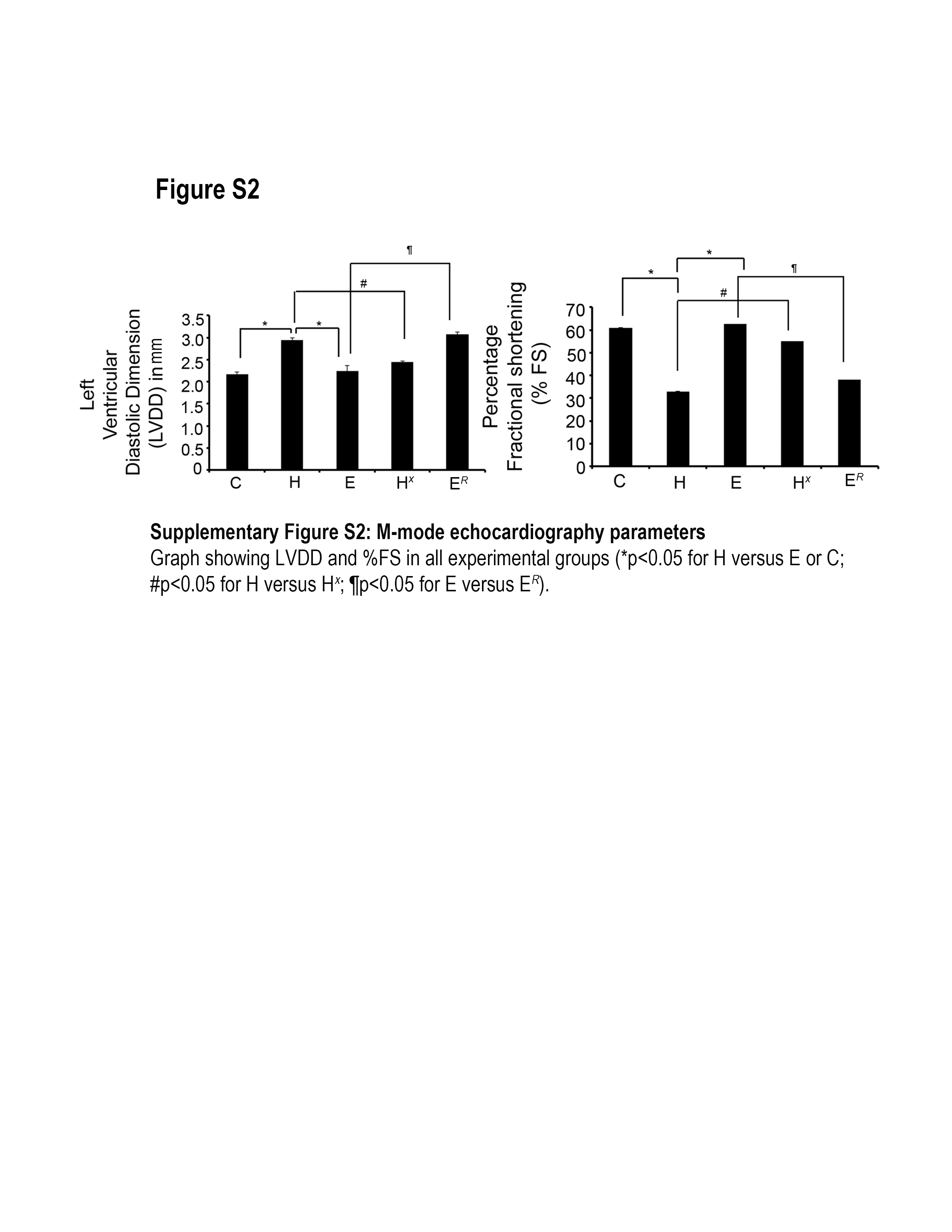

Supplement: Figure S2 — M-mode echocardiography parameters. Graph showing LVDD and %FS in all experimental groups (*p<0.05 for H versus E or C; #p<0.05 for H versus HX; ¶p<0.05 for E versus ER). (TIF) [file pone.0104711.s002.tif]

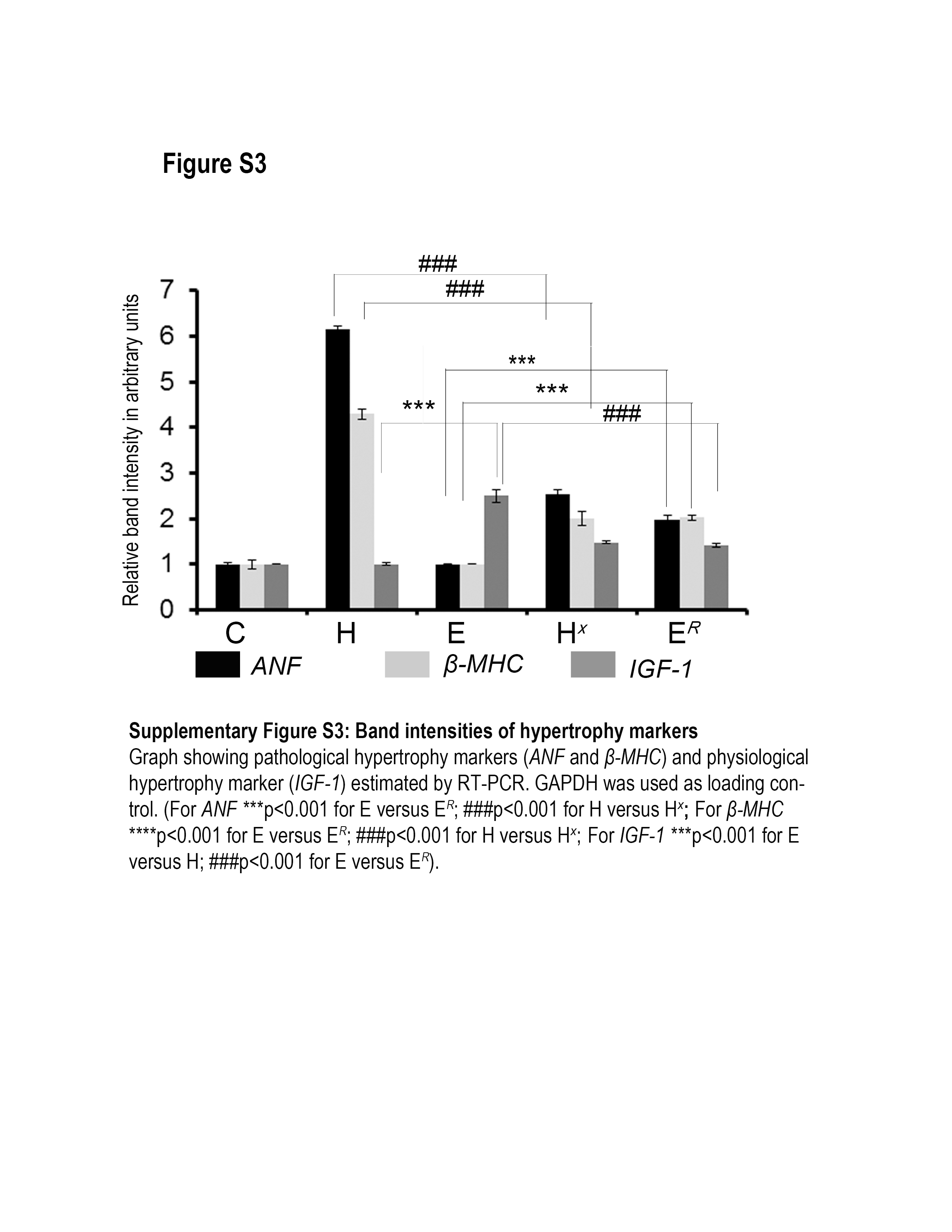

Supplement: Figure S3 — Band intensities of hypertrophy markers. Graph showing pathological hypertrophy markers (ANF and β-MHC) and physiological hypertrophy marker (IGF-1) estimated by RT-PCR. GAPDH was used as loading control. (For ANF ***p<0.001 for E versus ER; ###p<0.001 for H versus HX; For β-MHC ****p<0.001 for E versus ER; ###p<0.001 for H versus HX; For IGF-1 ***p<0.001 for E versus H; ###p<0.001 for E versus ER). (TIF) [file pone.0104711.s003.tif]

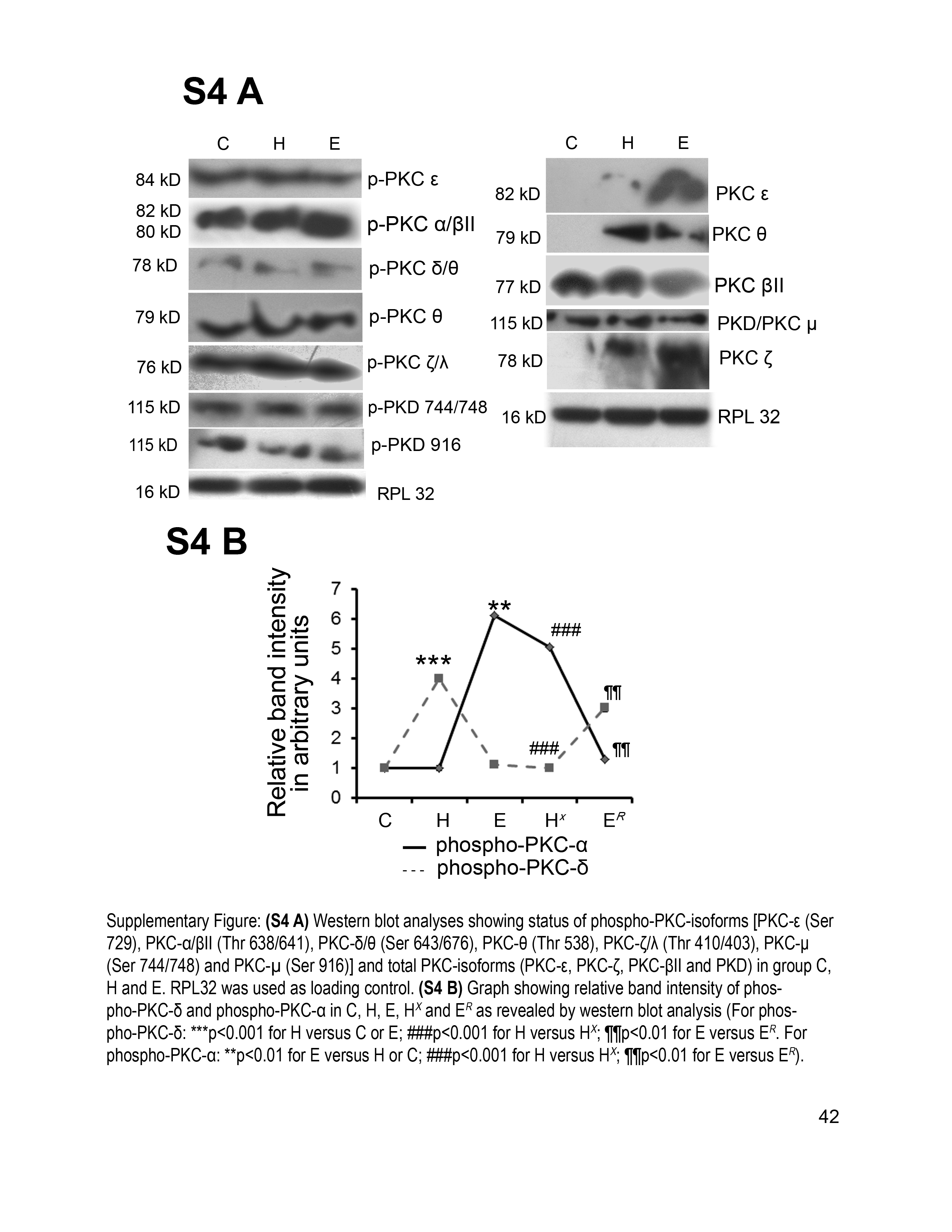

Supplement: Figure S4 — Expression level of different PKC-isoforms. (A) Western blot analyses showing status of phospho-PKC-isoforms [PKC-ε (Ser 729), PKC-α/βII (Thr 638/641), PKC-δ/θ (Ser 643/676), PKC-θ (Thr 538), PKC-ζ/λ (Thr 410/403), PKC-µ (Ser 744/748) and PKC-µ (Ser 916)] and total PKC-isoforms (PKC-ε, PKC-ζ, PKC-βII and PKD) in group C, H and E. RPL32 was used as loading control. (B) Graph showing relative band intensity of phospho-PKC-δ and phospho-PKC-α in C, H, E, HX and ER as revealed by western blot analysis (For phospho-PKC-δ: ***p<0.001 for H versus C or E; ###p<0.001 for H versus HX; ¶¶p<0.01 for E versus ER. For phospho-PKC-α: **p<0.01 for E versus H or C; ###p<0.001 for H versus HX; ¶¶p<0.01 for E versus ER). (TIF) [file pone.0104711.s004.tif]

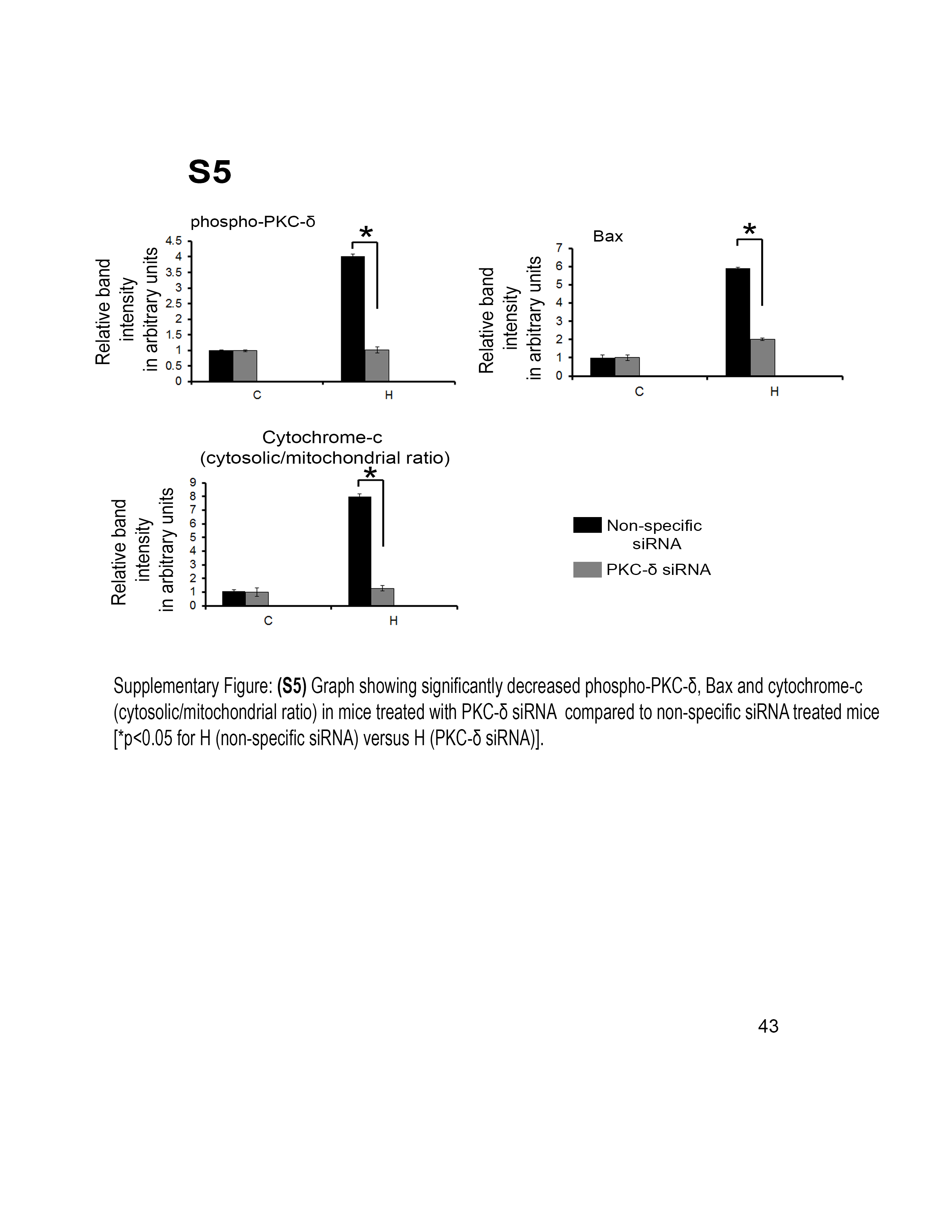

Supplement: Figure S5 — Silencing of PKC-δ results in down regulation of apoptotic markers. Graph showing significantly decreased phospho-PKC-δ, Bax and cytochrome-c (cytosolic/mitochondrial ratio) in mice treated with PKC-δ siRNA compared to nonspecific siRNA treated mice [*p<0.05 for H (nonspecific siRNA) versus H (PKC-δ siRNA)]. (TIF) [file pone.0104711.s005.tif]

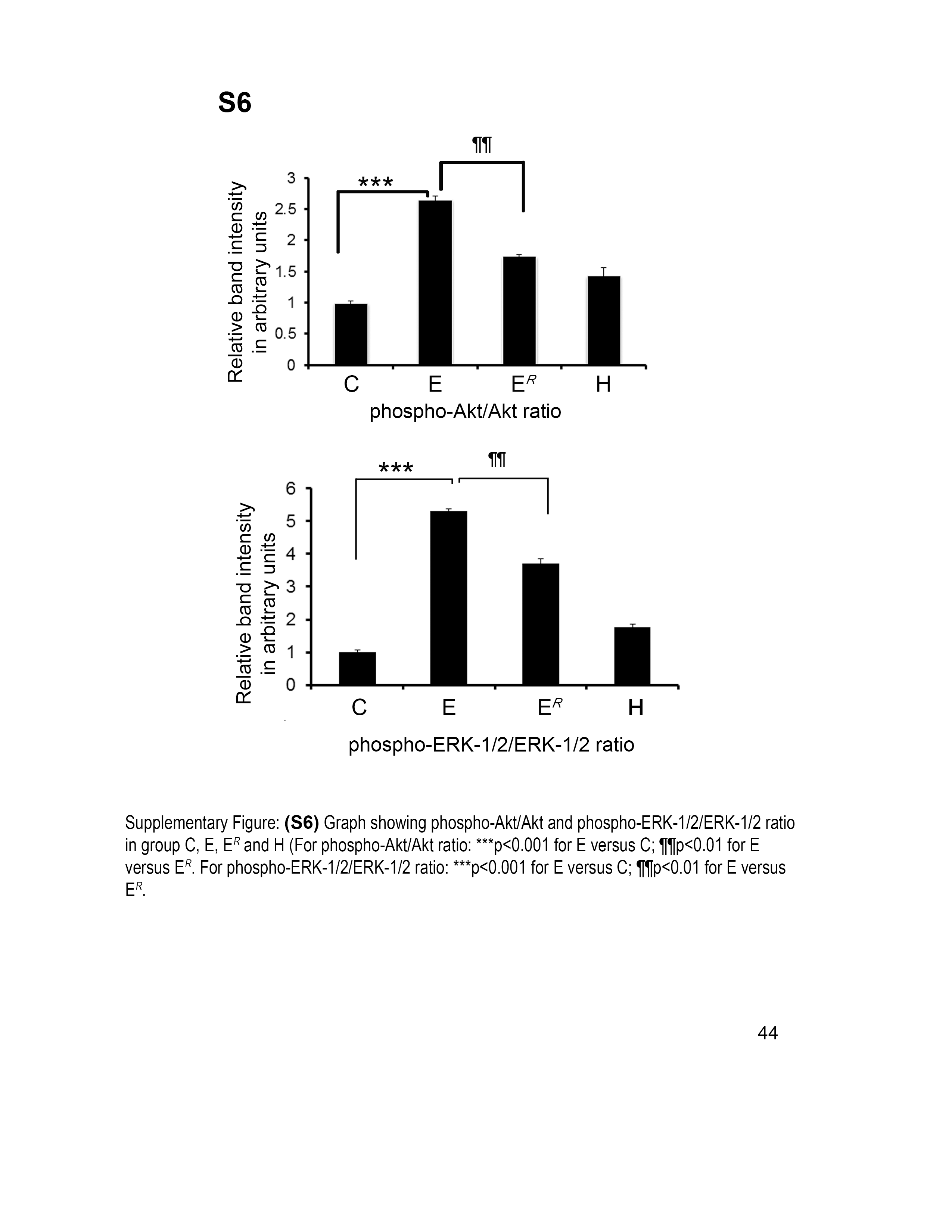

Supplement: Figure S6 — Relative expression of Akt and ERK-1/2. Graph showing phospho-Akt/Akt and phospho-ERK-1/2/ERK-1/2 ratio in group C, E, ER and H (For phospho-Akt/Akt ratio: ***p<0.001 for E versus C; ¶¶p<0.01 for E versus ER. For phospho-ERK-1/2/ERK-1/2 ratio: ***p<0.001 for E versus C; ¶¶p<0.01 for E versus ER. (TIF) [file pone.0104711.s006.tif]

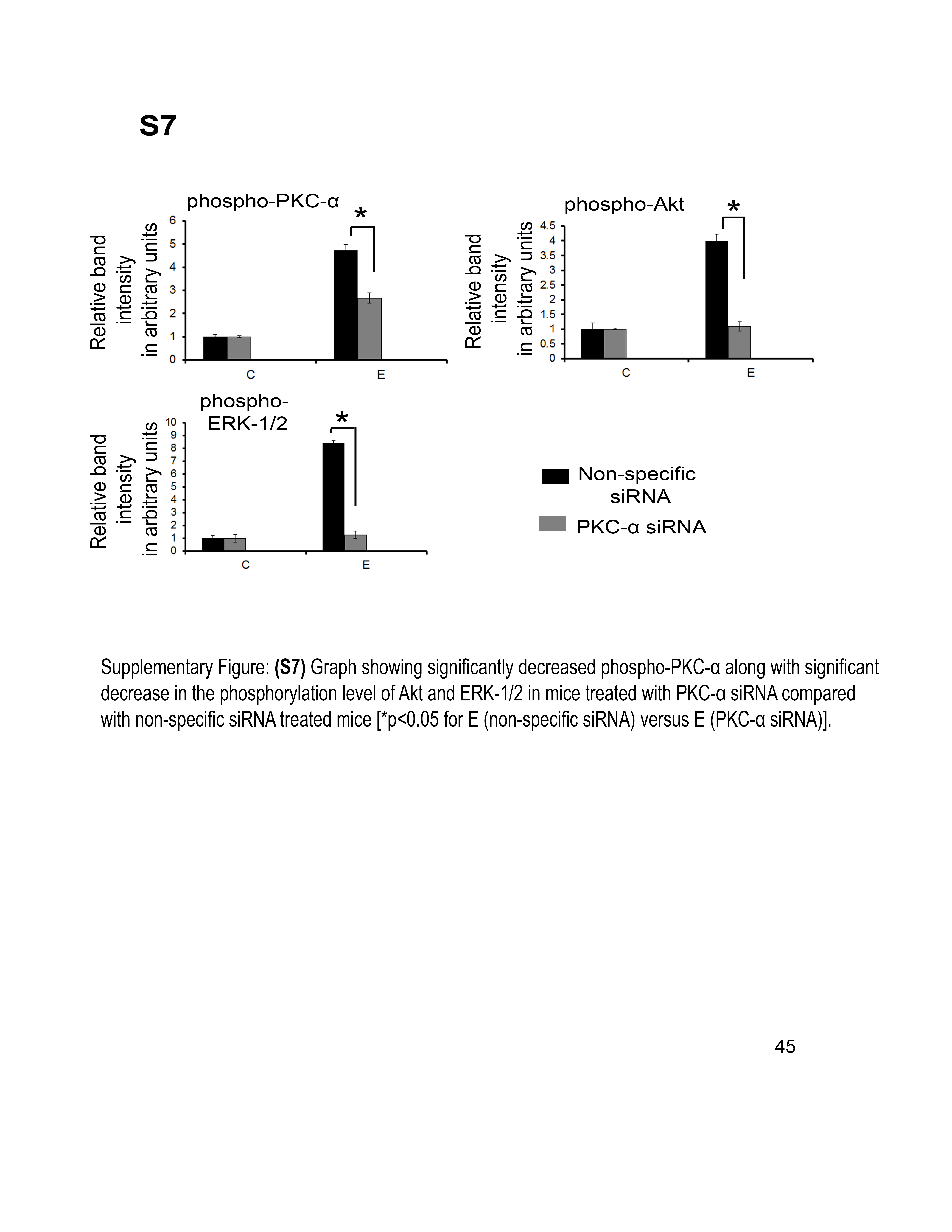

Supplement: Figure S7 — Silencing of PKC-α results in down regulation of prosurvival markers. Graph showing significantly decreased phospho-PKC-α along with significant decrease in the phosphorylation level of Akt and ERK-1/2 in mice treated with PKC-α siRNA compared with nonspecific siRNA treated mice [*p<0.05 for E (nonspecific siRNA) versus E (PKC-α siRNA)]. (TIF) [file pone.0104711.s007.tif]

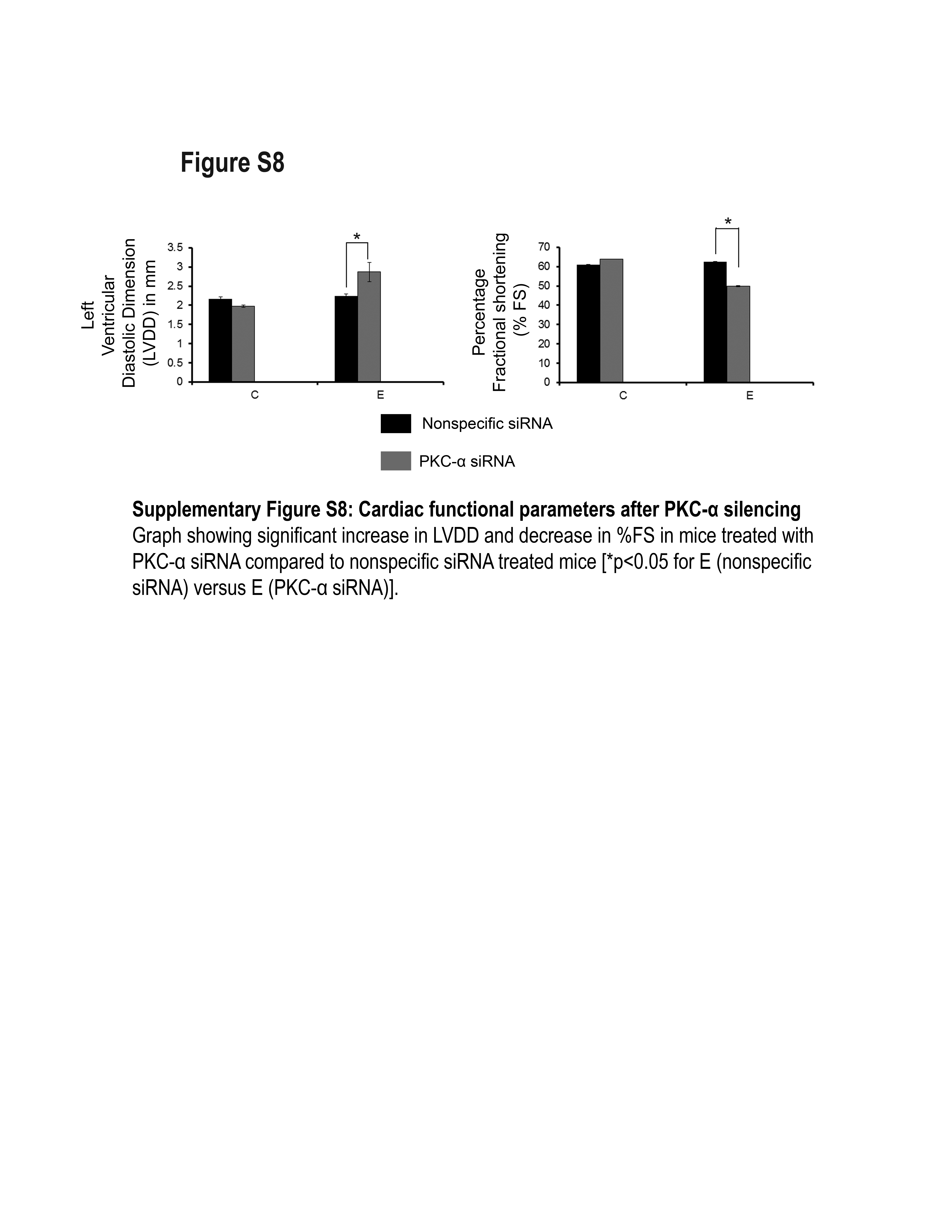

Supplement: Figure S8 — Cardiac functional parameters after PKC-α silencing. Graph showing significant increase in LVDD and decrease in %FS in mice treated with PKC-α siRNA compared to nonspecific siRNA treated mice [*p<0.05 for E (nonspecific siRNA) versus E (PKC-α siRNA)]. (TIF) [file pone.0104711.s008.tif]

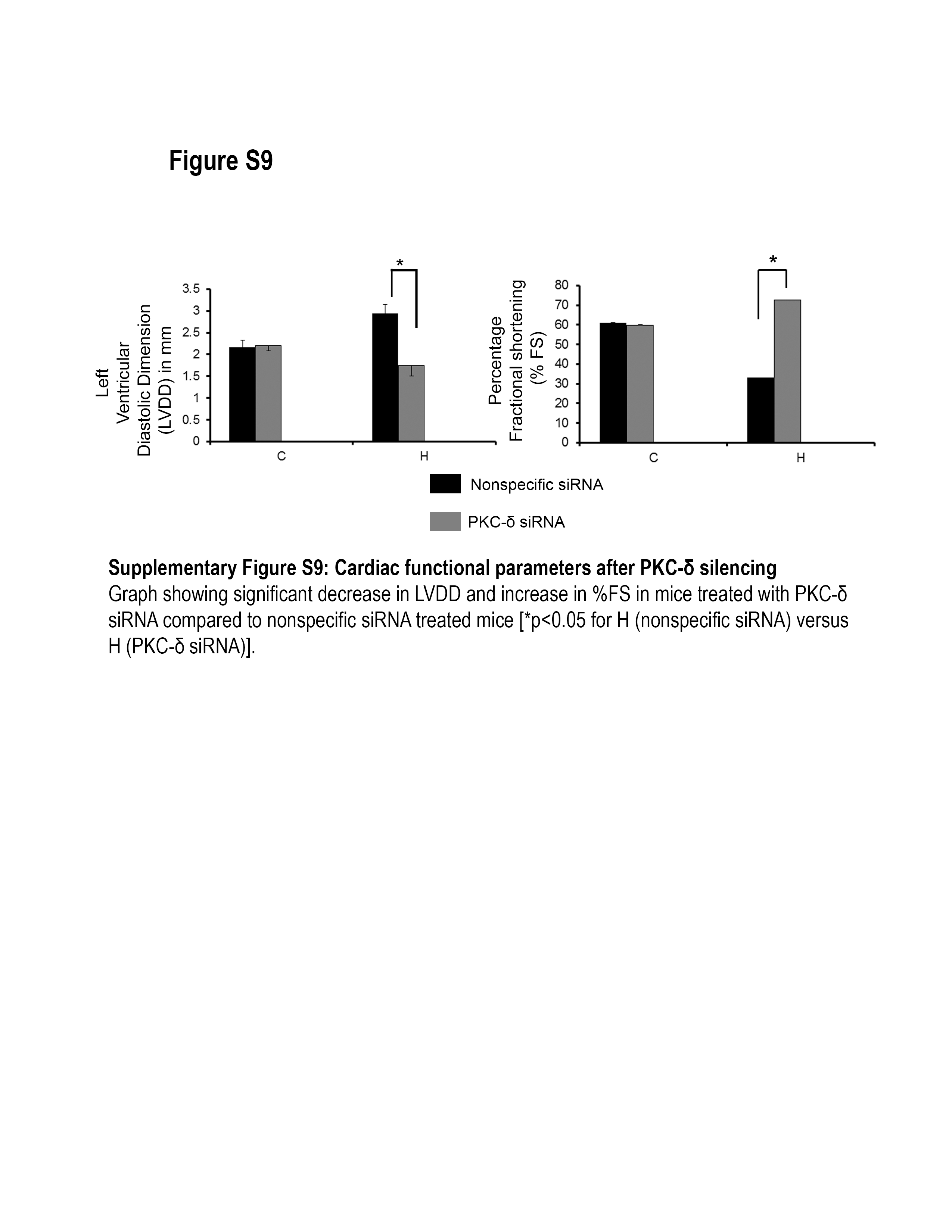

Supplement: Figure S9 — Cardiac functional parameters after PKC-δ silencing. Graph showing significant decrease in LVDD and increase in %FS in mice treated with PKC-δ siRNA compared to nonspecific siRNA treated mice [*p<0.05 for H (nonspecific siRNA) versus H (PKC-δ siRNA)]. (TIF) [file pone.0104711.s009.tif]
